# A genome-wide RNA interference screening reveals protectiveness of *SNX5* knockdown in a Parkinson’s disease cell model

**DOI:** 10.1186/s40035-025-00486-5

**Published:** 2025-06-03

**Authors:** Matthias Höllerhage, Linghan Duan, Oscar Wing Ho Chua, Claudia Moebius, Svenja H. Bothe, Kristina Losse, Rebecca Kotzur, Kristina Lau, Franziska Hopfner, Franziska Richter, Christian Wahl-Schott, Marc Bickle, Günter U. Höglinger

**Affiliations:** 1 Department of Neurology, Hannover Medical School, Carl-Neuberg-Str. 1, 30625 Hannover, Germany; 2 Center for Systems Neuroscience, Hannover, Germany; 3 HT-Technology Development Studio, Max Planck Institute of Molecular Cell Biology and Genetics, Dresden, Germany; 4 Department of Pharmacology, Toxicology and Pharmacy, University of Veterinary Medicine Hannover, Hannover, Germany; 5 Department of Neurology, LMU University Hospital, Ludwig-Maximilians-Universität (LMU), Munich, Germany; 6 The Institute of Cardiovascular Physiology and Pathophysiology, Ludwig-Maximilians-Universität (LMU), Munich, Germany; 7 German Center for Neurodegenerative Diseases (DZNE), Munich, Germany; 8 Munich Cluster for Systems Neurology (SyNergy), Munich, Germany

**Keywords:** Genome-wide RNAi screening, Parkinson’s disease, Alpha-synuclein, Retromer, SNX5, Trans-Golgi network

## Abstract

**Background:**

Alpha-synuclein (αSyn) is a major player in the pathophysiology of synucleinopathies, which include Parkinson’s disease, dementia with Lewy bodies, and multiple system atrophy. To date, there is no disease-modifying therapy available for these synucleinopathies. Furthermore, the intracellular mechanisms by which αSyn confers toxicity are not yet fully understood. Therefore, it is of utmost importance to investigate the pathophysiology of αSyn-induced toxicity in order to identify novel molecular targets for the development of disease-modifying therapies.

**Methods:**

We performed the first genome-wide siRNA modifier screening in a human postmitotic neuronal cell model using αSyn-induced toxicity as a read-out. In a multi-step approach, we identified several genes, whose knockdown protected against αSyn-induced toxicity. The main hit was further validated by different methods, including immunofluorescence microscopy, qPCR, and Western blot. Furthermore, the main finding was confirmed in mouse primary neurons.

**Results:**

The highest protection was achieved by knockdown of *SNX5*, which encodes the sorting nexin 5 (SNX5) protein, a component of the retromer complex. The protective efficacy of *SNX5* knockdown was confirmed with an independent siRNA system. The protective effect of *SNX5* knockdown was further confirmed in primary neurons from transgenic mice, where the knockdown of *SNX5* led to amelioration of decrease in synchrony that was observed in untreated and control-siRNA-treated cells. SNX5 protein is a component of the SNX-BAR (Bin/Amphiphysin/Rvs) heterodimer, which is part of the retromer complex. Extracellular αSyn and overexpression of intracellular αSyn led to fragmentation of the trans-Golgi network, which was prevented by *SNX5* knockdown that led to confinement of αSyn in early endosomes.

**Conclusion:**

In summary, our data suggest that SNX5 plays an important role in the trafficking and toxicity of αSyn. Therefore, SNX5 appears to be a target of therapeutic intervention for synucleinopathies.

**Supplementary Information:**

The online version contains supplementary material available at 10.1186/s40035-025-00486-5.

## Background

Synucleinopathies are a group of neurodegenerative diseases defined by the presence of intracellular proteinaceous inclusions consisting mainly of aggregated alpha-synuclein (αSyn). In cases of PD and dementia with Lewy bodies, these αSyn aggregates are found in neurons and designated as Lewy bodies [1]. Multiple system atrophy is characterized by αSyn aggregates, called glial cytoplasmic inclusions, in oligodendrocytes [2]. Physiologically, αSyn is a small, unfolded and soluble protein of 140 amino acids. However, in synucleinopathies, αSyn aggregates and confers neuronal toxicity. In PD, the typical motor symptoms (bradykinesia, rigidity, and tremor) are caused by the death of dopaminergic neurons in the substantia nigra pars compacta. It is widely accepted that αSyn plays a role in synapses [3]. The mechanisms of how αSyn becomes toxic are not fully understood. However, a common assumption suggests that small oligomeric species occurring in the aggregation process are toxic for neuronal cells [3]. Furthermore, the release of αSyn into the extracellular space and the uptake of αSyn species by neighboring cells are believed to play a role in cell-to-cell spreading of αSyn pathology throughout the brain, which is considered a mechanism involved in the progression of the disease [4]. In order to investigate αSyn pathophysiology, we established a model in differentiated, postmitotic dopaminergic Lund human mesecenphalic (LUHMES) neurons [5], in which moderate overexpression of human wild-type αSyn leads to ~ 50% of cell death, accompanied by the occurrence of a 37-kDa oligomeric species of αSyn that was not present in control cells [6]. In this model, we have previously investigated intracellular mechanisms involved in αSyn degradation and identified protective interventions [7–9]. In the present work, we performed a genome-wide siRNA screening in this model to identify genes whose knockdown protects against αSyn-induced toxicity. The most interesting hit was sorting nexin 5 (SNX5), a member of the retromer complex, which is a cargo recognition complex involved in endosome sorting and endosome–trans Golgi network (TGN) trafficking [10].

Since mutations in vacuolar protein sorting ortholog 35 (VPS35), another component of the retromer complex, are associated with hereditary forms of PD [11], and endocytosis and endosomal trafficking have been linked to uptake and release of αSyn [12], our focus was to investigate the involvement of SNX5 in the regulation and trafficking of αSyn and how *SNX5* gene knockdown protects dopaminergic neurons from αSyn-induced cytotoxicity.

The aim of this study was to perform a genome-wide siRNA screening in order to identify novel molecular targets for a potentially disease-modifying therapy for neurodegenerative synucleinopathies, and perform a deeper characterization of the main hit from this screening.

## Methods

### LUHMES cell culture

LUHMES cells [5] were cultured at 37 °C with 5% CO_2_ and 100% humidity, as previously described [8, 9]. Briefly, for proliferation, LUHMES cells were cultured in growth medium (GM) consisting of DMEM/F12 (Sigma-Aldrich, St. Louis, MO), 1% N2 supplement (Life Technologies, Carlsbad, CA) and 0.04 µg/mL basic fibroblast growth factor (PeproTech, Rocky Hill, CT) in cell culture flasks (Nunc, Thermo Fisher Scientific, Waltham, MA). Before cell seeding, the flask was pre-coated with poly-*L*-ornithine (PLO) (0.1 mg/mL, incubation overnight at 37 °C) and washed three times with phosphate buffered saline (PBS; Life Technologies). For differentiation, the cells were seeded in differentiation medium (DM) consisting of DMEM/F12, 1% N2 supplement, 1 µg/mL tetracycline (Sigma-Aldrich), 0.49 µg/mL dibutyryl cyclic adenosine monophosphate (Sigma-Aldrich), and 2 ng/mL glial cell-derived neurotrophic factor (R&D Systems, Minneapolis, MN). The experiments were conducted on flasks or multi-well plates (Nunc; ThermoFisher Scientific) double-coated with PLO as described above followed by a second coating with 5 µg/mL fibronectin (R&D Systems) through incubation overnight at 37 °C and washed once with PBS.

## Primary neuron preparation

Male mouse pups from the transgenic Thy1-αSyn (Line 61) mouse model [13, 14] were sacrificed on postnatal days D1–3 by decapitation and brains were extracted. The brains were then dissected into small pieces in 4 °C isolation medium containing Hibernate™-A medium (Thermo Fisher Scientific), 1% B-27™ Plus Supplement (Thermo Fisher Scientific), and 0.5 mmol/L *L*-Glutamine (Sigma Aldrich, G3126). After 2 washes with cold isolation medium, warm Papain (Merck Millipore, Burlington, MA, P4762) Solution (2 mg/mL in HEPES buffer) was added and incubated for 24 min at 37 °C with 5% CO_2_, stirred gently every 5 min. To stop the digestion, warm isolation medium was added and the brains were centrifuged at 300 × *g* (Heraeus Multifuge 1S-R; ThermoFisher Scientific; acceleration set to 7 and deceleration set to 5) for 10 min at room temperature (RT). After stepwise homogenization in warm isolation medium, the tissue was passed through a 100-µm strainer and centrifuged at 300 × *g* (acceleration and deceleration as described above) for 10 min at RT. The pelleted cells were resuspended in cultivation medium containing Neurobasal-A medium (Thermo Fisher Scientific), 1% B-27™ Plus Supplement, 0.5 mmol/L *L*-Glutamine, and 1% Penicillin/Streptomycin (Thermo Fisher Scientific) and counted.

## Primary cell culture

Primary mouse neurons were cultivated in Neurobasal medium (Thermo Fisher Scientific) supplemented with B-27 (50 ×; 2%; Thermo Fisher Scientific), *L*-Glutamine (0.5 nmol/L; Sigma Aldrich) and Penicillin/Streptomycin (100 ×; 1%; Thermo Fisher) on cell culture dishes or multiwell microelectrode array (MEA) plates coated with Poly-*D*-Lysine (50 µg/ml; Sigma Aldrich) at 37 °C with 5% CO_2_. Cells were cultivated for 21 days with medium change twice a week until maximum synchrony was reached.

## Adenoviral transduction

Adenoviral vectors of serotype 5 (BioFocus, Charles River Laboratories Nederland B.V., Leiden, Netherlands) were used to overexpress human wild-type αSyn or green fluorescent protein (GFP) under a cytomegalovirus promotor. The vectors were not produced within this project but provided by the manufacturer. Briefly, the backbone of the vectors was human mastadenovirus serotype 5 (reference sequence: M73260). In these vectors, nucleotides that contain the E1 region had been deleted and replaced by a polymerase 2 expressing cassette comprising: CMV enhancer/promotor derived from human cytomegaloviruses IE1 gene promotor region, a synthetic region containing polylinker sequences with cDNA sequences inserted into this polylinker, and a SV40 poly(A) signal derived from SV40. Furthermore, the vectors contained an E2 A deletion, in which the E2 A coding region was replaced by a synthetic oligonucleotide. These modifications were performed by the manufacturer to disable the vectors from amplification outside of packaging cells used in the manufacturing process. The αSyn-expressing vectors were provided as 25 µL master aliquots with a titer of 1.7 × 10^10^ vectors per milliliter. These were diluted in PBS (total 500 µL or 20-fold dilution) to achieve a concentration of 8.5 × 10^8^ vectors per milliliter. The GFP-expressing vectors were provided as 10 µL master aliquot with 5.7 × 10^10^ vectors per milliliter. These were further diluted in PBS (total 670 µL or 67-fold dilution) to achieve the same concentration of 8.5 × 10^8^ vectors per milliliter. Prior to the transduction, these virus dilutions were further diluted in the cell culture medium to achieve the required multiplicity of infection (MOI). In the high-content screening, in which the cells were seeded in GM, a MOI of 5 (5 viral vectors per seeded cell, 2.5-fold higher than used for transduction of cells seeded in DM; see below) was used to account for proliferation of the cells on the day after seeding. Twenty-four hours after transduction, the remaining viral vectors were removed by three washes with PBS. In the experiments investigating pathophysiology, the cells were transduced with the vectors two days after seeding in DM with a MOI of 2 (2 viral vectors per seeded cell). The use of lower MOI was because cells that were seeded in DM do not proliferate anymore.

## Genome-wide RNA interference screening

In the high-throughput screening, the cells were prepared as in a previous compound screening study [9]. Briefly, cells were seeded in double-coated flasks in GM with medium changed to DM after 24 h. One day after initiation of differentiation, the cells were transduced with adenoviral vectors to overexpress human αSyn. One day later, the remaining virus particles were removed by rinsing the cells with PBS and then the cells were detached using trypsin–EDTA (5 min incubation at 37 °C; Sigma-Aldrich) and reseeded in double-coated 384-well multi-well plates one day after transduction. By doing so, we achieved maximal homogeneity throughout the screening plates. After seeding in the multi-well plates, the cells were treated with different endoribonuclease-prepared small interfering RNAs (esiRNAs). esiRNAs are an RNA interference (RNAi) system with high efficiency and small off-target effects [15, 16]. Cell death was quantified as the percentage of cells with propidium iodide (PI) incorporation. Cell survival was quantified as cells without PI incorporation. PI is an intercalating compound that is actively removed from living cells and therefore accumulates only in dead cells. For the quantification, the cells were incubated with 4 μg/mL PI (Sigma-Aldrich) and 2 μg/mL Hoechst 33342 to stain all cell nuclei. Imaging was performed after 15 min with an Opera High Content Screening System (Perkin Elmer, Waltham, MA).

In the whole screening, a genome-wide library containing esiRNAs against 16,744 genes was tested. Cells transfected with an esiRNA against αSyn were used as a positive control (best survival). Cells transfected with an esiRNA against firefly luciferase (F-Luc), and mock transfected cells, were used as negative control. In the primary screening, the survival of the cells after transfection with all individual esiRNAs was compared to the average survival of the whole library and Z-scores were calculated (Z-statistics). Primary hits were defined as esiRNAs that led to a survival Z-score > 2.4 in at least two screening runs compared to the whole library. In total, 46,627 individual experiments were performed.

In the secondary screening, the cells were seeded, transduced with adenoviral vectors, transfected with the esiRNA, and imaged in the same way. In this screening, three runs were performed and an ANOVA followed by a Dunnett’s *post-hoc* test was used to compare the survival rate. All esiRNAs that led to a significantly better survival than the F-Luc esiRNA (*P*-value < 0.05) were considered as secondary hits.

In the tertiary screening, the survival of the cells transfected with these esiRNAs was investigated again in GFP-expressing cells and the survival was compared to the survival of αSyn-overexpressing cells. Those esiRNAs that specifically protected against αSyn as determined by multiple T-tests were considered as final hits.

## siRNA transfection

The cells were transfected with either MISSION® esiRNA (Sigma-Aldrich) at a concentration of 200 nmol/L or siPOOL siRNA (SiTOOLs Biotech, Munich, Germany) at a concentration of 5 nmol/L. In preliminary experiments, siPOOl siRNAs were also used at 10 nmol/L. However, we did not observe a higher knockdown efficacy (Supplementary file 1: Fig. S1) and therefore, 5 nmol/L was used for all further experiments. The concentrations were chosen based on the recommendations by the manufacturers. Before distribution, the integrity and purity of individual siPOOLs were verified by high-performance liquid chromatography by the manufacturer. Before transfection, the siRNAs were mixed with OptiMEM medium (Thermo) and Lipofectamine RNAiMax (2 µL/mL; Thermo Fisher Scientific) and incubated at RT for 20 min. The sequences of used siPOOL siRNAs are shown in Supplementary file 2.

## Quantitative real-time polymerase chain reactions (qPCR)

For qPCR, RNA was extracted using the RNeasy Mini kit (Qiagen, Venlo, Netherlands) and RNA concentration was determined with a NanoDrop 2000 (Thermo Fisher Scientific) spectrometer. Subsequently, the RNA samples were reverse transcribed into cDNA using the iScript cDNA Synthesis kit (Bio-Rad Laboratories, Hercules, CA). qPCR was performed with a CFX96 Touch Real-Time PCR Detection System (Bio-Rad Laboratories), using SYBR Select (Thermo Fisher) as dye with the following cycling conditions: an initial denaturation at 95 °C for 10 min, followed by 40 cycles of 95 °C for 15 s and 60 °C for 1 min. The following primers were used for LUHMES cells: *SNX1* forward: AAGCACTCTCAGAATGGCTTC, reverse: CGGCCCTCCGTTTTTCAAG; *SNX2* forward: GGGAAGCCCACCGACTTTG, reverse: GGCCATTGGAGTTTGCACTAATA; *SNX5* forward: TCTGTATCTGTGGACCTGAATGT, reverse: GTGGGCAGTGTGGTCTTTGT); *SNX6* forward: TCTTTGAGCACGAACGAACA, reverse: CATCAGCAGCACTTTTGTGAG; *VPS35* forward: GTCAAGTCATTTCCTCAGTCCAG, reverse: CCCCTCAAGGGATGTTGCAC. The following primers were used for primary neurons: *SNX5* forward: AGGACCGCAGCAAGTTAAGA; reverse: TGTGGACAGTGTGGTCTTGG.

## Western blot

For Western blot, whole cell extracts were collected using M-PER lysis buffer (Thermo Fisher Scientific) supplemented with a protease inhibitor and phosphatase inhibitor cocktail (Merck Millipore) on ice. After centrifugation (15,000 × *g* for 10 min at 4 °C), the supernatant was collected and protein content was measured using the BCA Protein assay (Thermo Fisher Scientific) according to the manufacturer’s instructions. Proteins (20 µg) in Laemmli Sample Buffer (Bio-Rad Laboratories) were loaded on 4%–12% bis–tris gels (Bio-Rad Laboratories) or 4%–15% Tris–glycine gels (Bio-Rad). After SDS-PAGE, the proteins were blotted onto methanol-activated polyvinylidene difluoride (PVDF) membranes (0.2 µm) with a Trans-Blot SD Semi-Dry Transfer Cell system (Bio-Rad Laboratories). After blotting, the membranes were fixed in 0.4% paraformaldehyde (PFA) for 30 min. After rinsing with tris-buffered saline with 0.05% Tween-20 (TBS-T, pH 7.4), the membranes were blocked with 3 × Rotiblock for 1 h at RT. Thereafter, the membranes were incubated with the primary antibodies in 1 × Rotiblock in TBS-T overnight at 4 °C, rinsed three times with TBS-T, followed by incubation with a corresponding horse radish peroxidase (HRP)-conjugated secondary antibody in 1 × Rotiblock in TBS-T for 2 h at RT. After rinsing three times with TBS-T, the membranes were incubated with Clarity ECL substrate (Bio-Rad) for 10–15 min at RT. Images were taken with a LI-COR Odyssey® Fc imaging system (LI-COR Biotechnology, Lincoln, NE). β-Actin or glyceraldehyde 3-phosphate dehydrogenase (GAPDH) served as a loading control.

The following primary antibodies were used: mouse anti-SNX1 (1:1000; Santa Cruz Biotechnology; Dallas, TX), mouse anti-SNX2 (1:1000; Santa Cruz Biotechnology), mouse anti-SNX5 (1:1000; Santa Cruz Biotechnology), mouse anti-SNX6 (1:1000; Santa Cruz Biotechnology), goat anti-VPS35 (1:1000; Abcam, Cambridge, UK), rabbit anti β-actin (Cell Signaling Technology), and rabbit anti-GAPDH (1:2000; Cell Signaling Technology). The following secondary antibodies were used: HRP-coupled anti-goat, -mouse, or -rabbit antibody (1:5000; Vector Laboratories, Burlingame, CA). A list of all antibodies used in the Western blot analysis is presented in Supplementary file 1: Table S1.

## Immunocytochemistry

Sterile glass coverslips (Bellco Glass, Vineland, NJ) in 24-well plates (Nunc) or ibidi dishes (ibidi, Gräfelfing, Germany) were double-coated as described above. For immunocytochemistry, the cells were fixed in 4% PFA for 30 min at RT followed by permeabilization in Triton X-100 (0.1%; Sigma-Aldrich) for 15 min at RT or Tween-20 (0.1%) for 5 min at RT. After permeabilization, the cells were washed three times with PBS and then blocked with normal horse serum (NHS; 5%; Vector Laboratories) for 1 h at RT or overnight at 4 °C. Thereafter, the cells were incubated with the primary antibodies in NHS (5%) overnight at 4 °C, washed three times with PBS followed by incubation with the secondary antibodies for 2 h at RT. Ten minutes before the end of the incubation period, DAPI (4′,6-diamidino-2-phenylindole) (1 μg/mL final concentration) was added to the cells. After three washes with PBS, images were obtained. The following primary antibodies were used: anti-tubulin III (1:000; Santa Cruz Biotechnology), rabbit anti-TGN46 (1:1000; Abcam), mouse anti-Rab5a (1:100; Santa Cruz Biotechnology), rabbit anti-Rab7 (1:100; Abcam), rabbit anti-LAMP1 (1:1000; Abcam), rabbit anti-LAMP2a (1:200; Abcam), rabbit anti-p62 (1:50; Abcam), rabbit anti-LC3B (1:400; Abcam), and rabbit anti-Rab11a (1:1000; Invitrogen). The following secondary antibodies were used: Alexa Fluor 488 donkey anti-mouse or anti-rabbit antibody (1:1000; Thermo Fisher Scientific), Alexa Fluor 594 donkey anti-mouse or anti-rabbit antibody (1:1000; Thermo Fisher Scientific). A list of all antibodies used for immunocytochemistry is presented in Additional file 1: Table S2.

## Staining of activated caspases 3/7 (CellEvent™ staining)

On day 8 in vitro (DIV8), the cell culture medium was discarded and cells were incubated with 1 µmol/L CellEvent™ (Thermo Fisher Scientifc, 1:1000 dilution) in pre-warmed Hanks'Balanced Salt Solution (HBSS) for 30 min at 37 °C and 5% CO_2_. CellEvent™ is a dye that specifically stains activated caspases 3/7 and is therefore used as a marker for apoptosis. After incubation, the solution was replaced with pre-warmed HBSS containing 2 µg/mL Hoechst 33342 (Thermo Fisher Scientific).

## Lactate dehydrogenase (LDH) release measurement

Six days after transduction, the cell culture medium was collected to quantify LDH that was released into the cell culture medium as a measure for cytotoxicity. Briefly, 30 µL of conditioned medium was mixed with a reaction buffer containing 74.24 mmol/L Tris/HCl, 185.6 mmol/L NaCl, 3.2 mmol/L pyruvate, and 4 mmol/L nicotinamide adenine dinucleotide (NADH) in water. To measure LDH release, NADH turnover was quantified by repeated measurements of the absorbance at 340 nm over 4 min with a FLUOstar Omega (BMG Labtech, Ortenberg, Germany).

## Bafilomycin assay

Bafilomycin A1 (Thermo Fisher, 18029722) was diluted in DMSO to a 100 µmol/L stock solution. Three days after siRNA transfection, primary neurons were treated with 50 µmol/L bafilomycin A1 dissolved in NBBGP – Cultivation medium. Control treatment was established with an equivalent amount of DMSO in the culture medium. The cells were incubated for 24 h. Within this timeframe the cells remained at 37 °C and 5% CO_2_.

## Multielectrode assay

Primary neurons were plated at a density of 8 × 10^5^ per well directly on top of the electrodes of a CytoView MEA 24 (Axion Biosystems, Atlanta, GA) plate. The plates were pre-coated with 50 µg/mL Poly-*D*-Lysine several days prior to seeding. The plates were transferred daily into the Maestro Edge MEA system (Axion Biosystems) for evaluation of cellular functions during maturation. After bafilomycin A1 application, the cells remained in the MEA for 24 h with hourly measurements at 37 °C with 5% CO_2_. Baseline measurements were performed directly before Bafilomycin A1 application. Data were initially analyzed with the AxIS Navigator (Axion Biosystems).

## Treatment with labeled αSyn

Recombinant monomeric αSyn (18 mg/mL) was incubated with ATTO-565-N-hydroxysuccinimidyl-ester (ATTO-TEC, Siegen, Germany) in a sodium bicarbonate buffer according to the manufacturer’s instructions to fluorescently label αSyn. After the incubation, the concentration of the resultant solution containing labelled αSyn was adjusted to 2 mg/mL and excess unbound dye was removed using Bio-Spin 6 size exclusion spin columns (Bio-Rad Laboratories). ATT565-αSyn was added to the LUHMES cells on DIV6 at a final concentration of 2 µmol/L. After 24 h, αSyn was removed by three washes with PBS. To remove labeled αSyn on the outside of the cells, the cells were incubated with trypsin–EDTA for 30 s at 37 °C followed by three washes with PBS.

## Microscopy and image analysis

All microscopy imaging was performed with a Leica DMi8 inverted fluorescence microscope (Leica Camera AG, Wetzlar, Germany) and Leica Application Suite (LAS) X software. The density of the neuronal network was determined in images stained with the anti-tubulin III antibody by measuring the total branch length as a measure for network density and the number of quadruple points as a measure for network complexity, using a modified version of the ‘neurite analyzer’ plugin [17] in Fiji (Fiji Is Just ImageJ) version 2.14.0 for Windows 64-bit (https://imagej.net/software/fiji/). An automatized quantification of the CellEvent™ signal intensity in the nuclei region was performed using Fiji.

To quantify the amount of αSyn inside and outside the TGN, a line was drawn across the TGN or Golgi region in each image using LAS X, and the software automatically generated an intensity profile. In each cell analyzed, four regions of interest (ROIs) lines were selected to acquire intensity profiles from both the TGN and adjacent areas. Quantitative analysis was conducted by measuring the area under the curve of the signal intensities inside and outside the TGN region using Fiji. A minimum of 50 cells were evaluated for each experimental condition.

To analyze the colocalization, ROIs were selected by drawing a line across the TGN or Golgi region and the degree of colocalization between αSyn and TGN was quantified using the Fiji software version 2.14.0 for Windows 64-bit with the JACop plugin, which calculated the thresholded Mander’ overlap coefficient (M1t), with thresholds set automatically by the plugin [18].

The different states of the TGN (normal, scattered, and fragmented) were microscopically determined as previously described [19]. A normal TGN has an intact ring shape. A scattered TGN shows a less intensely stained ring shape with some fragments separated from the ring. A fragmented TGN does not show a ring shape, but fragments visible in the cytoplasm.

## Statistical analysis

Statistical analysis was performed using GraphPad Prism 10.0 (GraphPad Software, La Jolla, CA). All datasets were tested for normality using the D'Agostino and Pearson omnibus normality test. Unless otherwise specified, one-way ANOVA analysis of variance was performed, followed by a Tukey's *post-hoc* test or pairwise comparisons between selected groups. *P* < 0.05 was considered as statistically significant.

## Results

### Genome-wide esiRNA screening identified *SNX5* knockdown as being protective against αSyn-induced toxicity

**Fig. 1:**
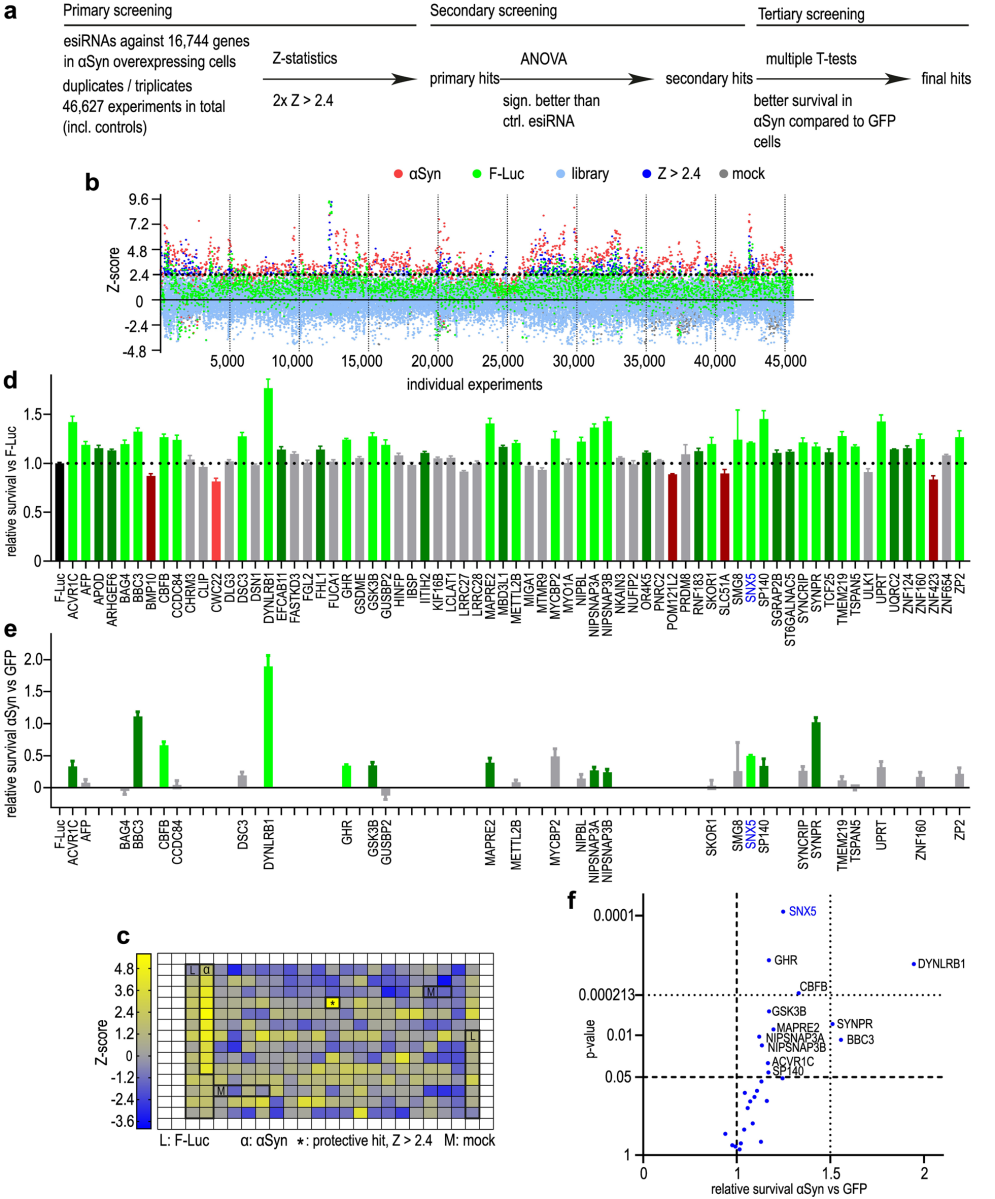
Genome-wide siRNA screening. **a** Flowchart of the screening process. Primary screening: Z-statistics (Z > 2.4 vs. library) to identify primary hits, defined as Z > 2.4 in at least 2 screening runs. Secondary screening: ANOVA analyses (vs. F-Luc as control esiRNA). Tertiary screening, final hits: multiple *T*-tests (compared to survival of GFP-overexpressing cells). **b** Z-scores of all experiments from the primary screening. Red dots: Z-scores of cells transfected with esiRNA against αSyn as positive control; green dots: Z-scores of cells transfected with esiRNA against luciferase (F-Luc) as negative control; light blue dots: Z-scores from the library; dark blue dots: Z-score > 2.4; grey dots: mock transfection. **c** Representative results from one screening plate. Thicker lines mark areas with controls (L: luciferase, M: mock transfection, α: αSyn transfection). **d** Secondary screening results: relative survival compared to the survival of controls (esiRNA against F-Luc). Green bars: increased survival (without correction for multiple testing); light green bars: hits after correction for multiple testing (Dunnett’s *post-hoc* test); red bars: reduced survival; light red bars: significant reduction of survival after correction for multiple testing; grey bars: no significant influence on survival. **e** Comparison of the survival between αSyn-overexpressing cells and GFP-expressing cells. Green bars: higher survival in αSyn-overexpressing cells with gene knockdown; light green bars: significantly higher survival after correction for multiple testing. **f** Volcano plot showing the relative survival of αSyn-overexpressing vs GFP-expressing cells. Lower dotted line: *P*-value < 0.05 in the individual *t*-tests; upper dotted line: *P*-value adjusted for multiple testing. SNX5 showed the smallest *P*-value of all screened genes

In postmitotic dopaminergic LUHMES neurons that degenerate upon moderate overexpression of human wild-type αSyn [6, 8, 9], we performed a genome-wide esiRNA screening with step-wise validation. In the primary screening, esiRNAs against 16,774 genes were tested for their protective efficacy against αSyn-induced toxicity in duplicates or triplicates, leading to 46,627 experiments in total. Primary hits were selected by a Z-statistic threshold. A secondary screening was conducted to confirm the protectiveness of the hits from the primary screening in comparison to a non-specific control siRNA. A third screening identified those esiRNAs that significantly protected against αSyn-induced toxicity compared to GFP-expressing cells to exclude non-specific effects on cell viability. A flowchart of the screening process is illustrated in Fig. 1a.

Positive hits were defined as esiRNAs leading to a better survival of αSyn-overexpressing cells with a z-Score > 2.4 above the mean survival. In the entire screening, in 1580 wells an esiRNA against αSyn was tested (positive control). Of these, 1246 led to a survival with a Z-score > 2.4 (true positive rate: 79%) and 334 did not meet hit criteria (false-negative rate: 21%). The average Z-score of survival of cells transfected with the esiRNA against αSyn was 3.3 ± 1.8 (standard deviation; SD). An esiRNA against firefly luciferase (F-Luc) was used as negative control. In total, 3964 wells were transfected with the F-Luc esiRNA, 3566 of which yielded a Z-score < 2.4 (true negative rate: 90%), whereas 398 showed a Z-score > 2.4 (false positive rate: 10%). The average Z-score of cells transfected with a F-Luc esiRNA was 1.2 ± 1.2, suggesting a minor unspecific and non-significant protective effect of the transfection procedure itself. In 1851 wells, a mock transfection was performed, yielding Z-scores < 2.4 in 1849 wells (true negative rate 100%) with an average Z-score of − 0.4 ± 1.0 (SD), showing no effect on cell survival. Results from the complete screening are illustrated in Fig. 1b. The results of an exemplary single plate are displayed in Fig. 1c.

In the primary screening, 80 of the 16,744 esiRNAs led to a higher cell survival in αSyn-overexpressing cells and were therefore selected as primary hits. Eleven of these esiRNAs were excluded because they could not be matched to a protein, were matched to a pseudogene, or were matched to a non-coding RNA. The remaining 69 esiRNAs were tested again in a secondary screening. This secondary screening was performed in order to confirm the protective efficacy of the identified primary hits. To reduce the number of false-positive hits, a more conservative statistical approach was followed in this secondary screening. Cell survival was compared to the survival of cells that had been transfected with a negative-control esiRNA against F-Luc and analyzed using an ANOVA followed by a Dunnett’s *post-hoc* test. After correction for multiple testing, 28 esiRNAs led to a significantly higher protection compared to the F-Luc esiRNA control and were therefore considered as confirmed secondary hits (Fig. 1d). In order to exclude unspecific protective effects on cell survival or adenoviral protein overexpression that was independent of αSyn-induced toxicity, we tested these 28 secondary hits again in αSyn-overexpressing cells and in cells that had been transduced with adenoviral vectors to express GFP as control protein. The survival rates of αSyn-overexpressing cells and GFP-expressing cells were compared using multiple *T*-tests. By doing so, we could identify genes whose knockdown specifically protected against αSyn-induced toxicity and had no effect on the general cell viability or on the adenoviral-mediated protein overexpression. Of the 28 secondary hits, 12 protected specifically against αSyn-induced toxicity (*T*-tests), and 4 of them (esiRNAs against *SNX5*, *DYNLRB1*, *CEFB*, and *GHR*) remained significant after correction for multiple testing (Fig. 1e, f**).** The esiRNA with the lowest *P* value targeted *SNX5*. Therefore, we decided to further elucidate the role of *SNX5* in our PD cell model.

## Confirmation of the protective efficacy of *SNX5* knockdown against αSyn-induced toxicity using siPOOL siRNAs

**Fig. 2:**
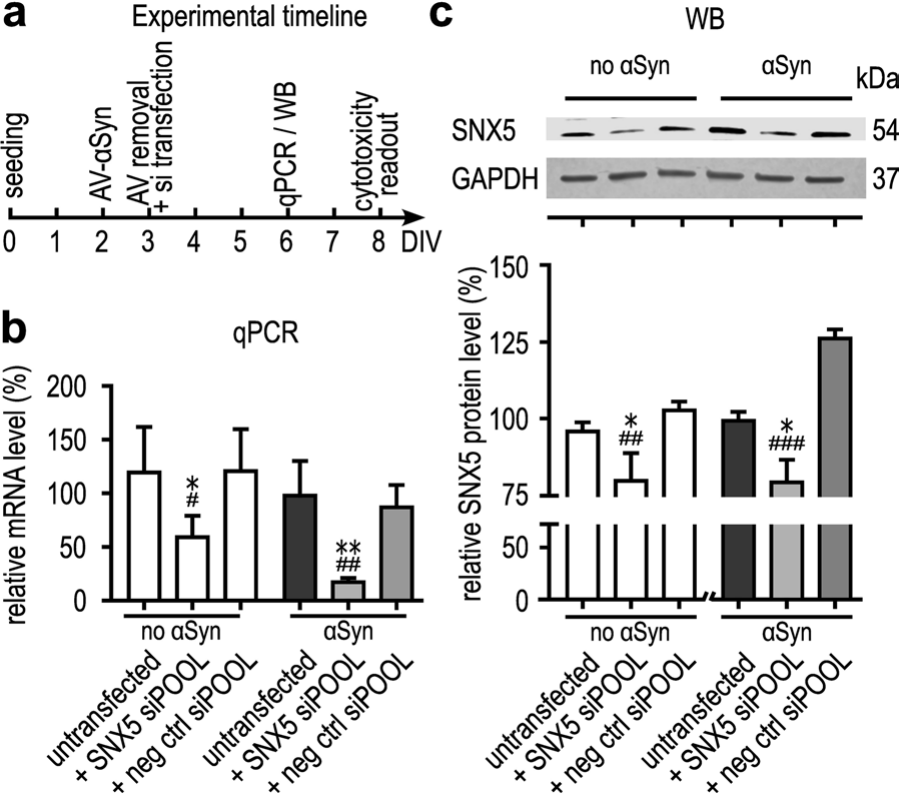
Validation of the knockdown efficacy of *SNX5* siPOOL siRNAs. **a** Experimental timeline of the validation experiment. AV: adenovirus, DIV: days in vitro.** b** Quantification of *SNX5* mRNA expression in control cells without αSyn overexpression and in αSyn-overexpressing cells by qPCR. Cells were transfected with siPOOL siRNA against *SNX5 *or a negative control siRNA, or untransfected. **c** Western blots for SNX5 and quantitation. The full Western blots are shown in Additional file 3. *P < 0.05, **P < 0.01 vs untransfected cells; ^#^*P* < 0.05, ^##^*P* < 0.01, ^###^*P* < 0.001 vs cells transfected with a negative control siRNA

After the screening process, we aimed to confirm the protective efficacy of *SNX5* knockdown against αSyn-induced toxicity with siPOOL siRNAs. LUHMES cells were transduced to overexpress αSyn and transfected with *SNX5*-siRNAs. The knockdown effect and the protective efficacy were determined four and six days after transduction, respectively (Fig. 2a). The siPOOL siRNA efficiently reduced *SNX5* mRNA levels by 93.1% ± 42.2% (*P* < 0.001). The protein level of SNX5 was also reduced after transfection with siPOOL siRNA against *SNX5*. The SNX5 protein level in cells treated with a negative control siPOOL siRNA was 126.7% ± 1.6% relative to that in the untransfected αSyn-overexpressing cells. After treatment with the siPOOL siRNA against *SNX5*, the SNX5 protein level was reduced to 80% ± 7.1% (*P* = 0.03 vs. untransfected cells, *P* < 0.0001 vs cells transfected with the negative control siPOOL siRNA) (Fig. 2b, c).

**Fig. 3:**
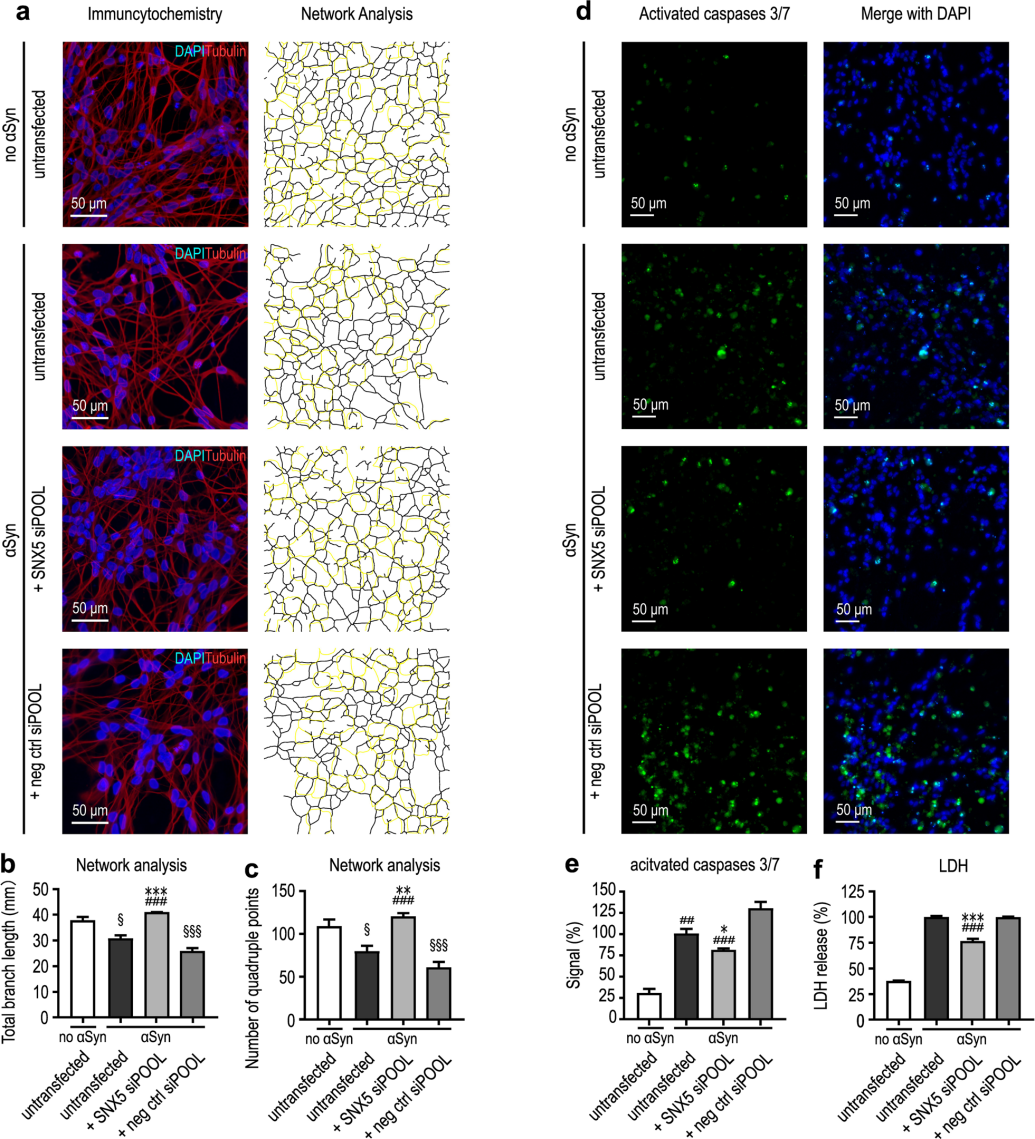
Validation of the protective efficacy of *SNX5* knockdown against αSyn-induced toxicity. **a** Immunofluorescence images of the neuronal network with an antibody against tubulin III (red) and nuclear co-staining with DAPI (blue) of untransduced cells (no αSyn; top panel), and αSyn-overexpressing cells (αSyn) without and with siRNA transfection. Panels on the right illustrate the neuronal network identified by the ‘neurite analyzer’ plugin in Fiji. The outline of the nuclei is highlighted in yellow. **b, c** Quantification of the total branch length (**b**) and the number of quadruple points as a measure for network complexity (**c**). **d** Immunofluorescence staining for activated caspases 3/7 (green; CellEvent) and nuclear with DAPI (blue). The conditions are the same as in panel **a**. **e** Quantification of the signal of activated caspases 3/7 after normalization to the signal in untransfected AV-αSyn cells. **f** Quantification of LDH released into the cell culture medium as a measure for cytotoxicity at the same conditions as in **a-e**. *P < 0.05, **P < 0.01, ***P < 0.001, vs untransfected AV- αSyn cells; ^##^*P* < 0.01, ^###^*P* < 0.001 vs AV- αSyn cells transfected with a negative control siRNA. ^§^*P* < 0.05, ^§§§^*P* < 0.001 vs. untransduced cells

Several analyses were performed to determine cell viability. We performed neuronal network analyses (Fig. 3a) and staining of active caspases 3/7 (Fig. 3d). Results of the network analysis showed that the total branch length was 37.2 ± 1.8 mm in untransduced cells, and reduced to 30.2 ± 1.7 mm upon αSyn overexpression (*P* < 0.05). However, knockdown of *SNX5* led to a protection of the neuronal network with total branch lengths of 40.4 ± 0.5 mm (*P* < 0.001 vs untransfected cells and cells transfected with control siRNA; Fig. 3b). In a similar manner, the number of quadruple points as a measure for network complexity was reduced upon αSyn overexpression (77.6 ± 7.7 vs. 107.6 ± 9.2 in untransduced control cells; *P* < 0.05). *SNX5* knockdown preserved the network complexity (121.3 ± 3.2 quadruple points), which was not observed with a negative control siRNA (Fig. 3c). Furthermore, we observed activation of caspases 3/7 upon αSyn overexpression in LUHMES cells (set to 100%), while in naïve cells, there was only a low signal of activated caspases 3/7 (30.7% ± 4.8%). Knockdown of *SNX5* using siPOOL siRNA led to a significant reduction of activated caspases 3/7 to 80.1% ± 1.6% (*P* = 0.03), whereas treatment with a negative control siPOOL siRNA led to an increase of activated caspases 3/7 (129.3% ± 7.4%) (Fig. 3d, e). Additionally, we quantified LDH release into the cell culture medium as a measure for cell death. In αSyn-overexpressing cells (set to 100%), knockdown of *SNX5* reduced the LDH release by 22.4% ± 6.1% (*P* < 0.001), which was not observed after transfection with a negative control siRNA (Fig. 3f). Together, these data confirmed that the knockdown of *SNX5* protected against the αSyn-induced toxicity. The total intracellular or extracellular αSyn levels were not affected by *SNX5* knockdown (Additional file 1: Fig. S2).

## Restoration of synchrony loss in primary neurons from αSyn transgenic mice upon *SNX5* knockdown

**Fig. 4:**
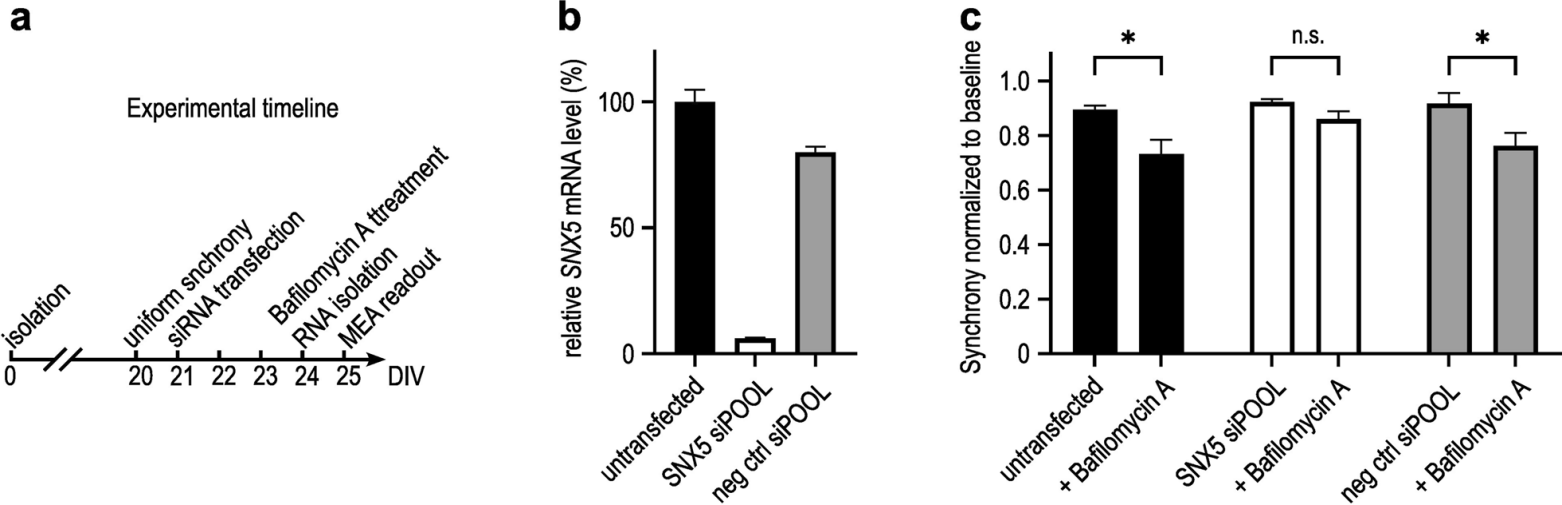
Restoration of synchrony loss in primary neurons from αSyn transgenic mice upon *SNX5* knockdown. **a** Experimental timeline of primary neuron isolation, maturation and siPOOL application in vitro; DIV, days in vitro. **b** Quantification of *SNX5* mRNA expression in primary neurons from the transgenic Thy1-αSyn (Line 61) mouse model. The cells were transfected with siPOOL siRNAs against *SNX5* or a negative control siRNA, or untransfected. **c** Multiwell microelectrode array (MEA) measurement of neuronal synchrony at 18 h after application of DMSO (left side bars) or 50 µmol/L bafilomycin A1 in DMSO of the same conditions as described in panel **b**. The graph shows results from 1 out of 3 representative technical replicates; bars represent mean ± SEM; n.s. = not significant, *P < 0.05; Two-way ANOVA with Sidak’s post-hoc test

Primary neurons derived from the Thy1-αSyn PD mouse model [13, 14] were cultivated until they reached maturity (Fig. 4a) indicated by a stable synchronous activity measured in the MEA system. After reaching maturation, the cells were transfected with the siPOOL siRNAs in the same fashion as for the LUHMES cells at 5 nmol/L. Both the siRNA against *SNX5* and the negative control siRNA were well tolerated and did not lead to cell death. Furthermore, we observed effective knockdown. The mRNA expression of *SNX5* was reduced to 6.0% ± 0.58% upon transfection with the siPOOL siRNA against *SNX5*, compared to 100% ± 8.4% in untransfected cells and 80.0% ± 0.38% in cells transfected with the negative control siRNA (Fig. 4b). This, combined with the high viability, indicated a good tolerability in the primary neurons. Three days post transfection with 5 nmol/L siPOOLs, the cells were exposed to culture medium containing 50 µmol/L bafilomycin A1 that inhibits autophagy to induce additional stress to these αSyn-overexpressing neurons, reducing their ability to form efficient networks. This was shown by a significant reduction of firing synchrony in response to bafilomycin A1, an observation not made in cells from wild-type mice (data not shown). In untransfected cells the synchrony index was reduced from 0.90 ± 0.01 to 0.73 ± 0.05 (*P* = 0.01) and in cells transfected with the negative control siRNA the synchrony index was reduced from 0.92 ± 0.04 to 0.76 ± 0.05 (*P* = 0.02) upon treatment with bafilomycin A1 (Fig. 4c). In contrast, primary cells with *SNX5* knockdown did not develop a significant reduction of firing synchrony. In cells with *SNX5* knockdown, the synchrony index was 0.92 ± 0.01 without and 0.86 ± 0.03 (*P* = 0.55) with treatment with bafilomycin A1. This indicates a neuroprotective effect of *SNX5* knockdown under these conditions (Fig. 4c).

## *SNX5* knockdown does not affect the expression of other retromer components

**Fig. 5:**
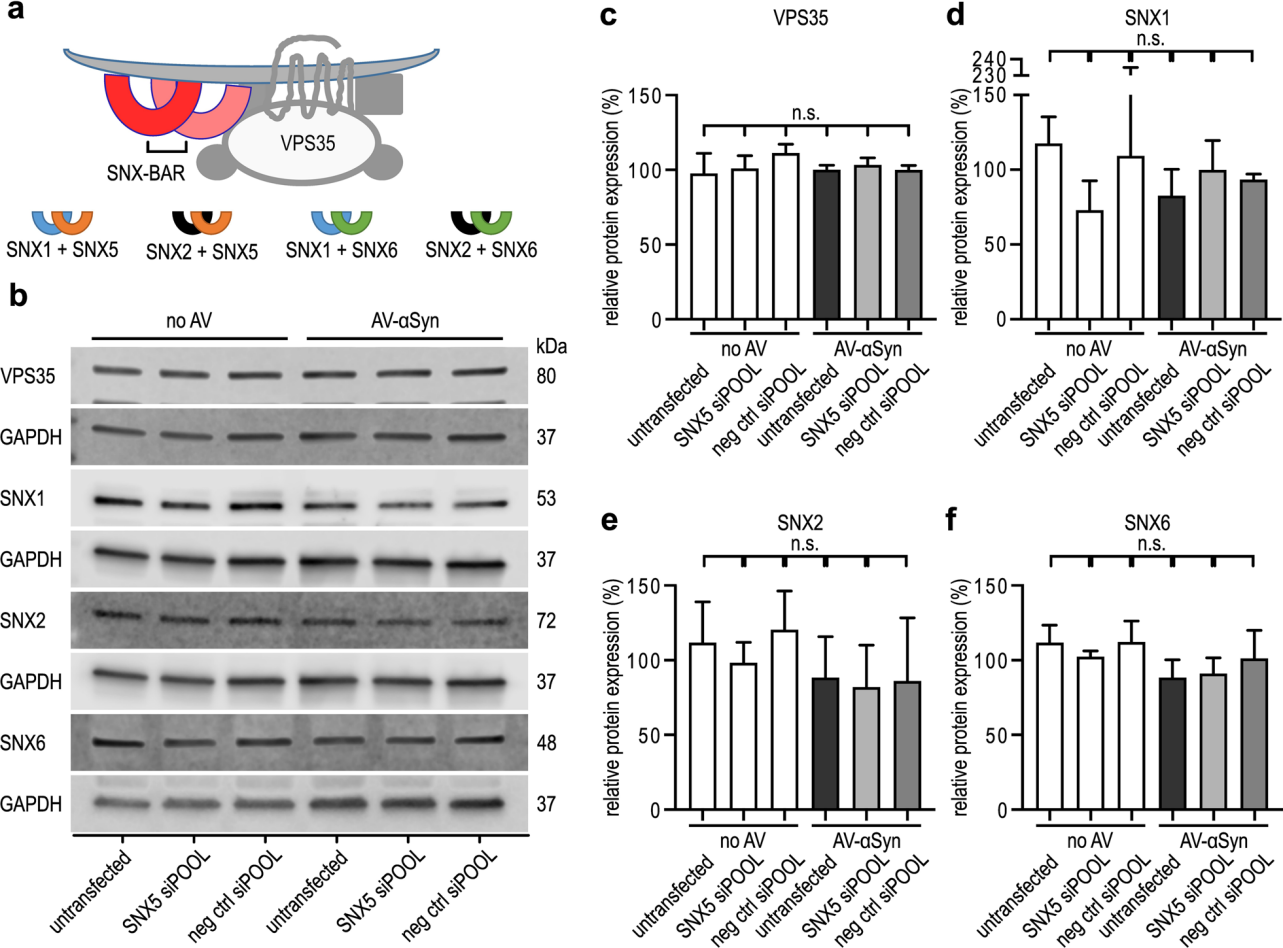
Effects of *SNX5* knockdown on the expression of other retromer components. **a** Schematic illustration of the retromer complex. The SNX-BAR heterodimers are composed of SNX1 (blue arches) or SNX2 (black arches) in combination with SNX5 (orange arches) or SNX6 (green arches). **b** Representative Western blots of other retromer components VPS35, SNX1, SNX2, and SNX6 in AV-αSyn cells and no-AV cells. The cells were either untransfected or transfected with siRNA against *SNX5* (SNX5 siRNA) or negative control siRNA (neg ctrl). Full Western blots are shown in Additional file 3: Fig. S6 b-e. **c-f** Quantification of the Western blot bands. Neither αSyn overexpression nor SNX5 knockdown (SNX5 siRNA) significantly altered the protein levels of the retromer components VPS35, SNX1, SNX2, and SNX6. Data are shown as mean ± SEM. n.s.: not significant, one-way ANOVA with Tukey’s *post-hoc* test

Since SNX5 protein is a component of the retromer complex (Fig. 5a) as part of the SNX-BAR heterodimers formed by SNX1/2 and SNX5/6, we investigated whether *SNX5* knockdown would lead to changes in the expression of other retromer components, as a possible compensatory mechanism. However, Western blot analysis did not reveal alterations of VPS35 or any of the other components of the SNX-BAR heterodimers (SNX1, SNX2, SNX6) upon *SNX5* knockdown (Fig. 5b–f). However, during investigation of the effects of knockdown of other SNXs, we found that *SNX6* knockdown led to a mild increase of LDH release, indicating a toxic effect of *SNX6* knockdown (Additional file 1: Fig. S3).

## Knockdown of *SNX5* prevents the transport of αSyn into the TGN

**Fig. 6:**
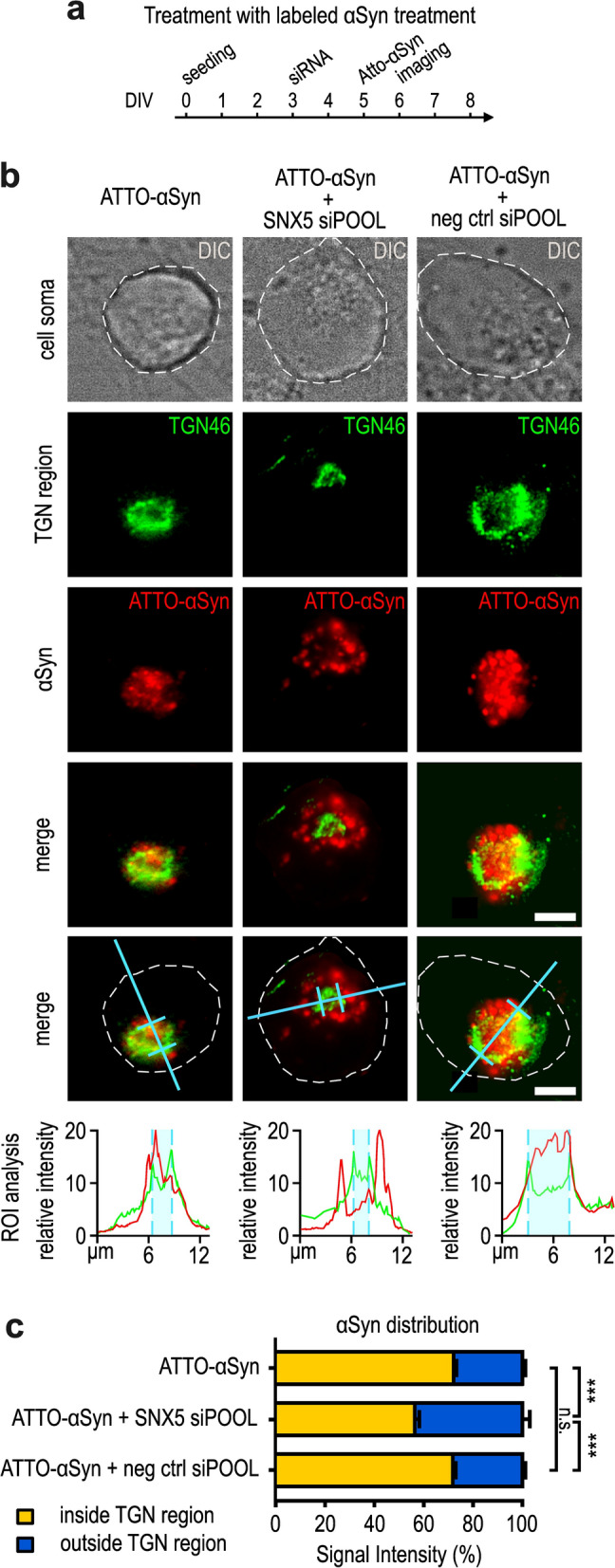
*SNX5* knockdown prevents internalization and accumulation of exogenous αSyn into the trans-Golgi network. **a** Experimental timeline of the treatment with fluorescently labelled αSyn. DIV, days in vitro. **b** Immunofluorescence staining of TGN46 (green) in cells treated with ATTO-565-labelled -αSyn (red). The turquoise lines indicate the location of intensity measurement. Lower panels: The turquoise area in the fluorescence intensity profiles indicate the outer margins of the TGN region, as defined by the TGN46 staining. The red line indicates the location of αSyn, both inside and outside of the TGN. After *SNX5* knockdown, αSyn was distributed outside the TGN as indicated by the red intensity signal (images in the middle), which was not the case with the negative control siRNA (right side images). Scale bar, 4 μm. **c** Quantification of the proportion of ATTO-αSyn inside and outside of the TGN region in the conditions shown in **b**. ***P < 0.001, ANOVA with Tukey’s *post-hoc* test. n.s. not significant

We next investigated the effect of *SNX5* knockdown on the transportation of αSyn within the cells (Fig. 6a). αSyn monomers were fluorescently labelled with ATTO-565 and added to the culture medium.

After fixing the cells, we performed immunocytochemistry staining with a TGN antibody (TGN46) and analyzed co-localization between αSyn and the TGN area. The TGN area was defined from the fluorescence signal of TGN46 staining (Fig. 6b, c). We then quantified the fluorescence signal of ATT-αSyn inside and outside this region, and confirmed that extracellularly added αSyn was indeed transported to the TGN region under normal conditions (Fig. 6b, left). Upon *SNX5* knockdown, the proportion of ATTO-αSyn inside the TGN region compared to outside the TGN region was shifted towards less αSyn inside the TGN region (Fig. 6b, middle). This effect was not observed upon transfection with a control siRNA (Fig. 6b, right). This suggests that *SNX5* knockdown leads to reduced trafficking of αSyn to the TGN.

## αSyn leads to TGN fragmentation which is ameliorated by *SNX5* knockdown

**Fig. 7:**
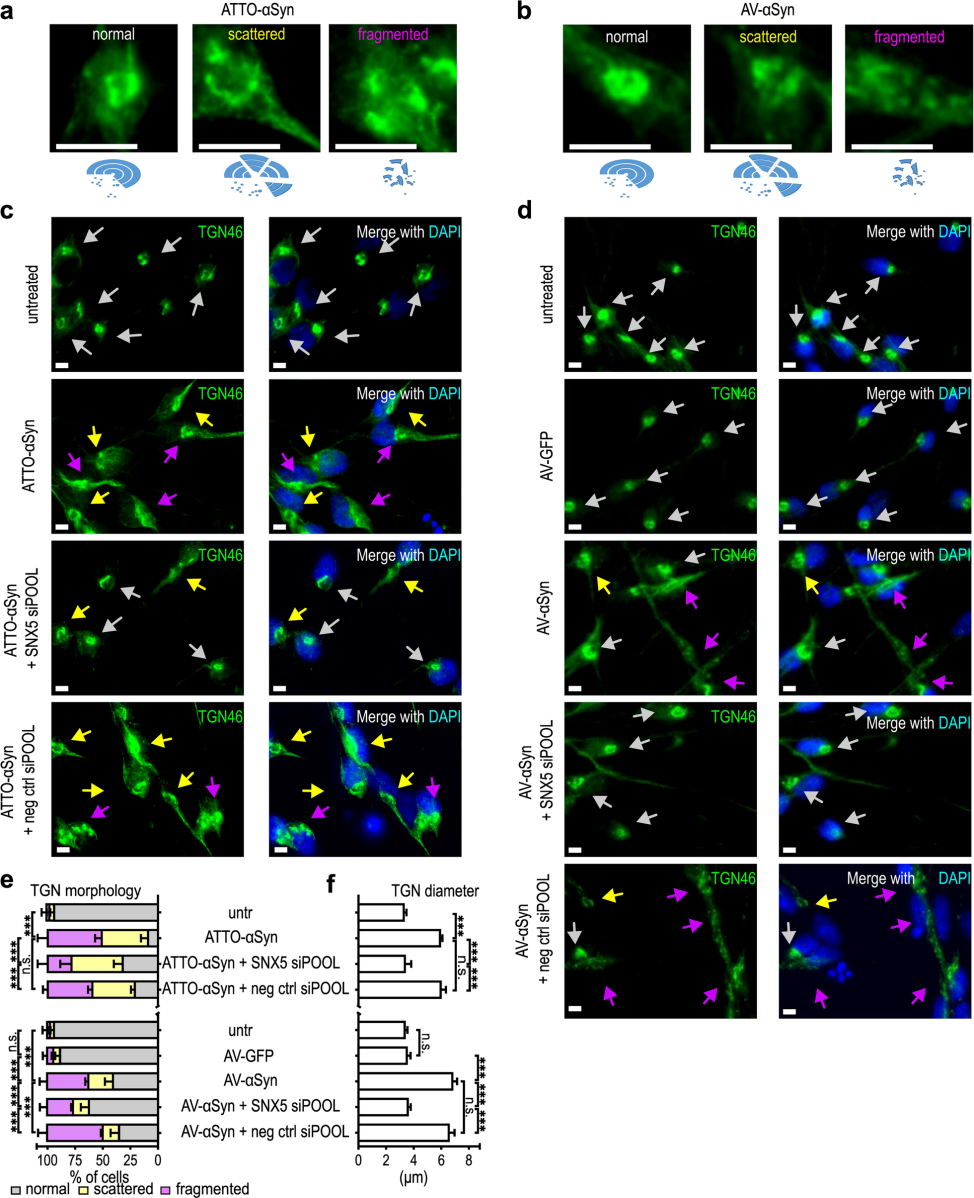
*SNX5* knockdown prevents TGN scattering and fragmentation in LUHMES cells. **a** Images of normal, scattered, and fragmented TGN morphology in LUHMES cells treated with ATTO-565-labeled recombinant αSyn monomers. Below are sketches of TGN morphology. All these morphologies were observed in αSyn-treated cells, but at varying degrees. **b** Images of normal, scattered, and fragmented TGN morphology in LUHMES cells transduced with αSyn-overexpressing adenoviral vectors (AV). Below are sketches of TGN morphology. **c** Representative images of staining for TGN46 in untreated cells, ATTO-αSyn-treated cells with no transfection, ATTO-αSyn-treated cells with SNX5 siRNA transfection, and ATTO-αSyn-treated cells with negative control (neg ctrl) siRNA transfection. The arrows indicate the different states of TGN morphology as illustrated in **a**. White arrows, normal morphology; yellow arrows, scattered morphology; purple arrows, fragmented morphology. A version of this panel that includes red staining of αSyn is shown in Fig. S5. **d** Representative images of staining for TGN46 in control cells (ctrl), AV-GFP cells, AV-αSyn cells, and AV-αSyn cells transfected with *SNX5* siRNA or negative control (neg ctrl) siRNA. **e** Percentage of cells with normal (grey), scattered (yellow), or fragmented (purple) TGN morphology in the experimental conditions of **c** and **d**. Exogenous αSyn (left) as well as adenoviral overexpressed αSyn (right) led to a higher percentage of scattered or fragmented TGNs, which was ameliorated by *SNX5* knockdown. The percentage of abnormal TGN (scattered or fragmented) was subtracted from 100% resulting in values for normal TGN. These were compared using ANOVA with Tukey's *post-hoc* test. **f** Quantification of the TGN diameter of normal and scattered TGN. Both exogenous αSyn (ATTO- αSyn) and adenoviral overexpressed αSyn (AV-αSyn) led to larger TGN sizes. ***P < 0.001. ANOVA with Tukey's *post-hoc* test. n.s. not significant

The morphology of the TGN can be classified into three states: normal, scattered, and fragmented [19]. The different TGN morphologies observed in LUHMES cells exposed to extracellular ATTO-αSyn are shown in Fig. 7a, and their relative quantitative frequency in Fig. 7e. The TGN morphologies in cells transduced to overexpress αSyn (AV-αSyn) are illustrated in Fig. 7b with quantitative data in Fig. 7e.

Then, we studied the effect of *SNX5* knockdown on TGN morphology in the presence of ATTO-αSyn (Fig. 7c) and AV-αSyn (Fig. 7d), using AV-overexpression of GFP as control protein.

Exposure to extracellular ATTO-αSyn or intracellular AV-αSyn overexpression, but not AV-GFP overexpression, led to a significant increase in scattered and fragmented TGN, as compared to untreated control cells. However, this effect was partially prevented by *SNX5*-knockdown, but not by a control siRNA (Fig. 7e).

Furthermore, ATTO-αSyn treatment led to an increase of the TGN diameter from 3.4 ± 0.1 μm (untreated controls) to 6.1 ± 0.2 μm (*P* < 0.001). Overexpression of αSyn led to an increase to 6.8 ± 0.2 μm (*P* < 0.001). However, knockdown of *SNX5* prevented this increase, reducing TGN diameter to 3.5 ± 0.2 μm in the presence of ATTO-αSyn and 3.6 ± 0.1 μm in AV-αSyn transduced cells, with no significant difference compared to untreated or AV-GFP-transduced cells (3.6 ± 0.1 µm; Fig. 7f). To confirm that TGN fragmentation leads to cytotoxicity, we treated the cells with brefeldin A, a compound known to disrupt the TGN [20], and observed increased activity of activated caspases 3/7 as an indicator for cytotoxicity (Additional file 1: Fig. S4).

## *SNX5* knockdown leads to increased levels of αSyn in early endosomes

To further investigate the mechanism of protection against αSyn-induced toxicity by *SNX5* knockdown, we examined the endosome-to-TGN pathway, the endosome-to-plasma membrane (PM; recycling endosome) pathway, the endosome-to-lysosome pathway, and the autophagosome-to-lysosome pathway.

**Fig. 8:**
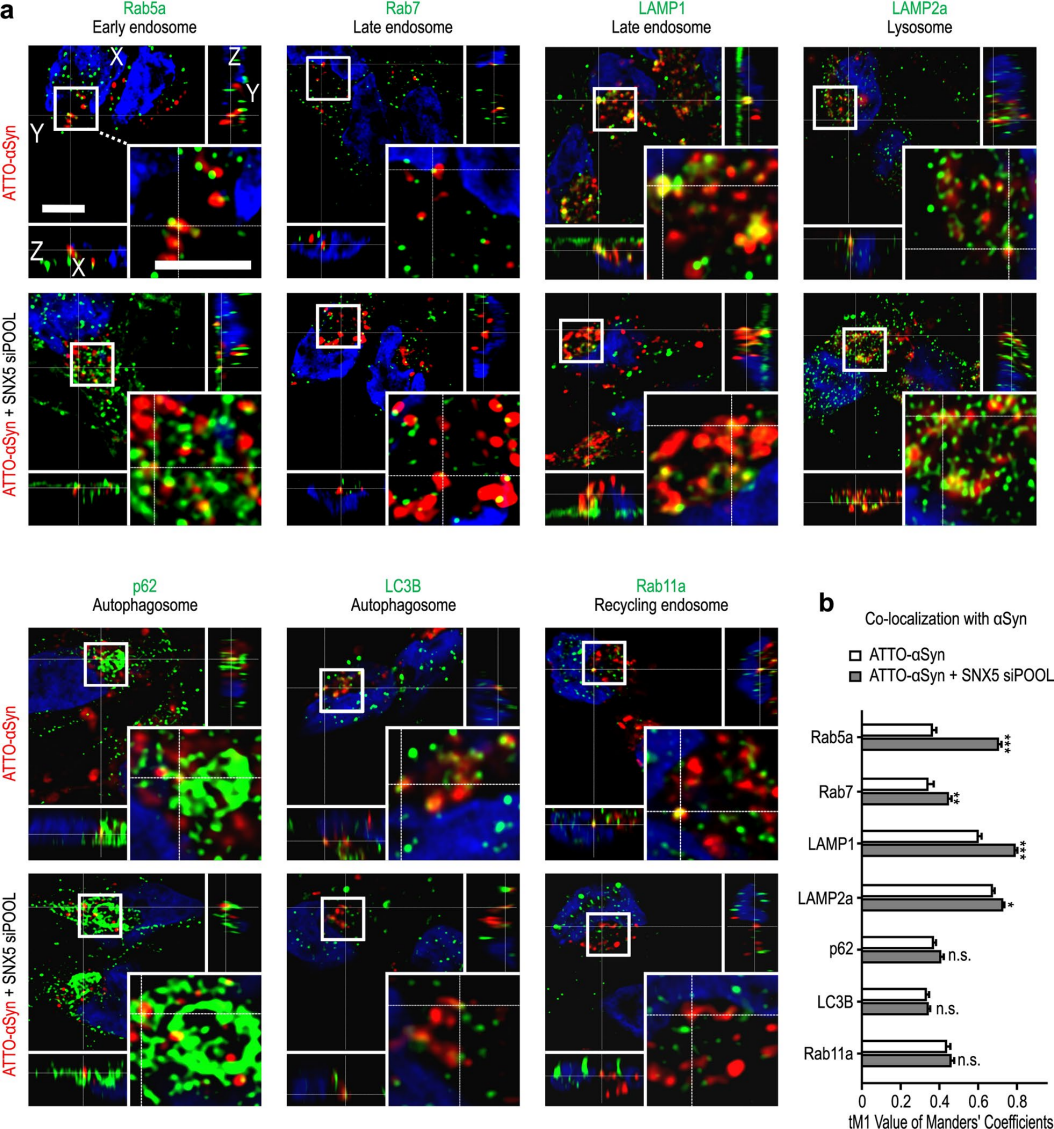
Co-localization of internalized αSyn with endocytosis markers. **a** Representative images of LUHMES cells treated with fluorescently labeled αSyn (ATTO-αSyn, red) with control siRNA transfection (top row of images), or after knockdown of *SNX5* (buttom row of images). A selected region of each image (white square) is shown in higher magnification in the right bottom corner. Scale bars: 4 µm. **b** Quantification of co-localization of exogenous αSyn with endocytosis markers. SNX5 knockdown led to increased co-localization between αSyn and early endosomes (Rba5a), late endosomes (Rab7, LAMP1), and lysosomes (LAMP2 A) compared to untransfected cells.Data are presented as mean ± SEM. *P < 0.05, **P < 0.01, ***P < 0.001 vs ATTO- αSyn, ^#^*P* < 0.05, ^##^*P* < 0.01, ^###^*P* < 0.001 vs ATTO- αSyn + SNX5 siRNA; n.s. not significant; one-way ANOVA with Tukey's *post-hoc* test

LUHMES cells were exposed to extracellular ATTO-αSyn either with or without *SNX5* knockdown, prior to fixation for immunocytochemistry. We used an antibody against Rab5a as a marker for early endosomes, antibodies against Rab7 and LAMP1 as markers for late endosomes, an antibody against LAMP2a as a marker for lysosomes, antibodies against p62 and LC3B as markers for autophagosomes, and an antibody against Rab11a as a marker for recycling endosomes (Fig. 8a). Manders’ overlap coefficient was determined to evaluate the degree of co-localization between αSyn and the different vesicular structures (Fig. 8b).

Under normal conditions, αSyn showed more co-localization (Manders’ overlap coefficient > 0.5) with endosomes and lysosomes than with autophagosomes or the recycling endosomes, suggesting that αSyn is mainly transported via the endosome-to-lysosome pathway and accumulates later in lysosomes under normal conditions.

**Fig. 9:**
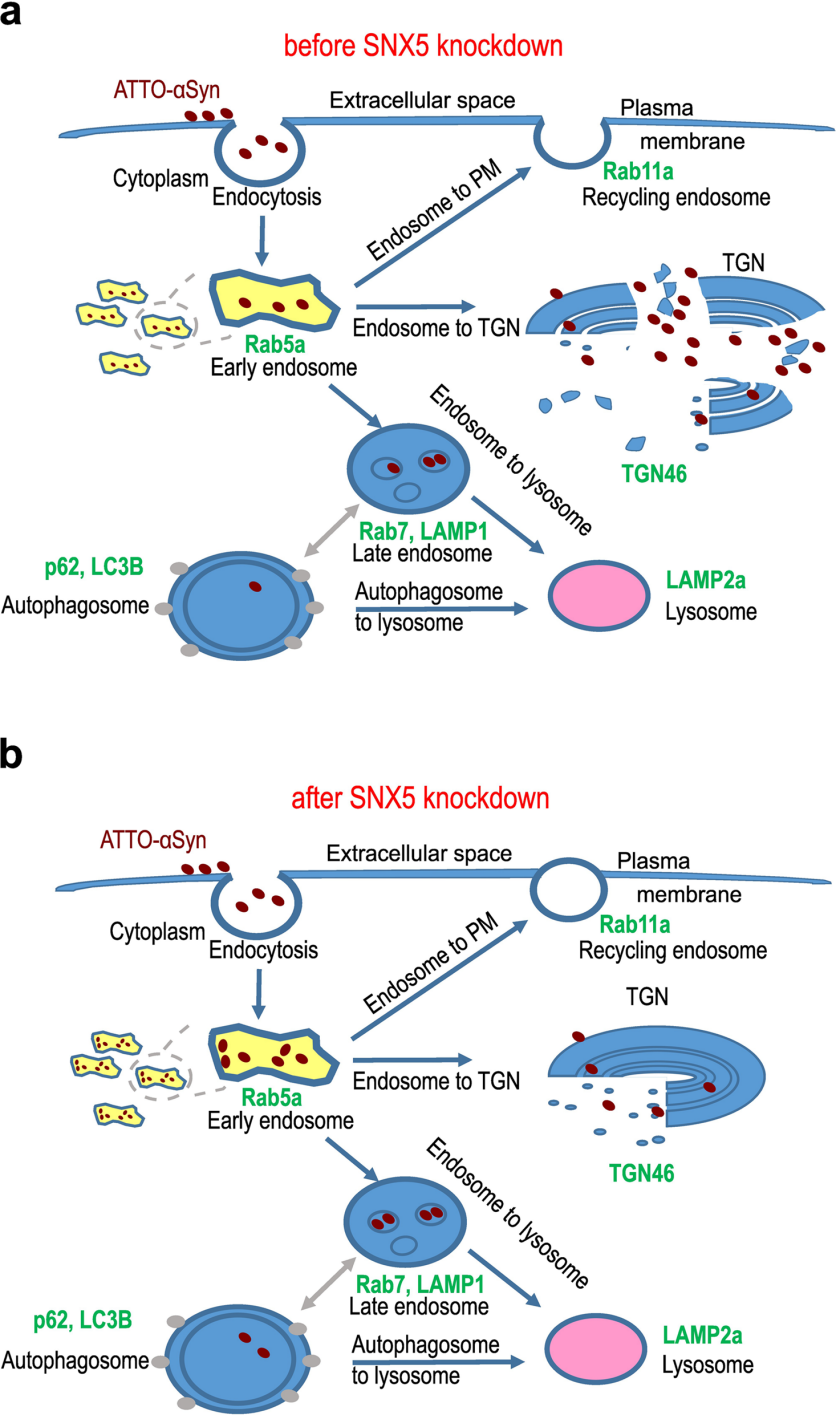
Transportation of αSyn before and after knockdown of *SNX5*. **a, b** Schematic illustration of possible endocytic pathways and the markers of different compartments used in our study (green). After endocytosis from the extracellular space, αSyn is transported into early endosomes (yellow; Rab5a). The early endosomes then either transport αSyn back to the plasma membrane (endosome to PM) in the form of recycling endosomes (Rab11), or to the trans-Golgi network (TGN; TGN46; endosome to TGN), or they form late endosomes (Rab7, LAMP1) to either transport αSyn directly to lysosomes (LAMP2a; endosome to lysosome) or first to autophagosomes (p62, LC3B) and then to lysosomes (autophagosome to lysosome). Before *SNX5* knockdown, more αSyn is transported to the TGN, while after *SNX5* knockdown less αSyn is transported to the TGN, but more to the early and late endosomes

*SNX5* knockdown led to a strong increase of co-localization of αSyn with early endosomes (Rab5), from 0.4 ± 0.02 to 0.77 ± 0.02 (*P* < 0.001), and a slight increase of co-localization with late endosome markers Rab7 (from 0.37 ± 0.04 to 0.49 ± 0.02; *P* < 0.001) and LAMP1 (from 0.66 ± 0.02 to 0.86 ± 0.02; *P* < 0.001). On the other hand, co-localization with autophagosomes and lysosomes was not altered (Fig. 8b). Together with the observation that *SNX5* knockdown led to reduced abundance of αSyn in the TGN (Fig. 6), these observations suggest that SNX5 facilitates αSyn trafficking from the early endosomes to the TGN (Fig. 9a) and that *SNX5* knockdown reduces this transport and leads to αSyn retention in early and late endosomes (Fig. 9b).

## Discussion

In the present work, we conducted a genome-wide multi-step RNAi screening in a PD human neuronal cell model to identify genes whose knockdown can protect against αSyn-induced toxicity. From 69 hits that were identified in the primary screening, 28 were confirmed in the secondary screening. After exclusion of genes whose knockdown unspecifically protected the cells as determined by their effect in GFP-expressing control cells, 12 genes remained, with 4 remaining significant after correction for multiple testing. Amongst these, SNX5, a component of the retromer complex, was the most promising candidate to follow-up. We first confirmed the protective efficacy of *SNX5* knockdown with a second RNAi system and by several assays. Furthermore, we used primary neurons derived from transgenic Thy1-αSyn (Line 61) mouse model [13, 14] to confirm the protective efficacy of *SNX5* knockdown. Upon *SNX5* knockdown, we observed a reduced loss of synchrony in these cells. *SNX5* knockdown increased the neuronal network density of αSyn-overexpressing LUHMES cells, reduced activation of caspases 3/7, and decreased LDH release (a measure for reduced cell membrane integrity). Since SNX5 is part of the SNX-BAR heterodimer within the retromer, we investigated if *SNX5* knockdown led to compensatory regulation of other retromer components, which was not the case. However, we found that in contrast to *SNX5* knockdown, the knockdown of SNX6, the alternative for SNX5 in a possible composition of SNX-BAR heterodimers, increased the αSyn-induced toxicity, suggesting that SNX5 is involved in the trafficking of toxic αSyn species. We found that αSyn overexpression and treatment with exogenous αSyn led to fragmentation of the TGN, and that *SNX5* knockdown prevented the TGN fragmentation by inhibiting the transport of αSyn to the TGN. Our findings suggest that SNX5 is involved in the regulation of the trafficking and toxicity of αSyn and could be a promising target for future development of neuroprotective therapies for PD and related synucleinopathies.

High-throughput RNAi screening is an effective way to identify new genes involved in the pathophysiology of distinct conditions in a hypothesis-free way. However, in the past, RNAi screenings that investigated modifiers of αSyn toxicity were performed only in subsets of the genome and/or in non-human cells (for review see [21]). To our knowledge, we present the first whole-genome RNAi screening of modifiers of αSyn toxicity. Furthermore, we utilized human postmitotic dopaminergic neurons (LUHMES cells), which very closely resemble the dopaminergic cells of the substantia nigra pars compacta, whose demise is responsible for the motor symptoms of PD patients. For the screening, we used a multistep approach to ensure that our final hits specifically modify the αSyn-induced toxicity.

On the one hand, SNX5 was the hit with the lowest *P*-value in the confirmatory screening process. On the other hand, as part of the SNX-BAR heterodimer which itself is part of the retromer, SNX5 is particularly interesting. It is known that the retromer plays a role in the pathophysiology of PD [22–24]. Mutations in VPS35, an important component of the retromer, are risk factors for PD, but the frequency of such mutations is not yet known [25, 26]. Moreover, the retromer is involved in multiple cellular processes including vesicular trafficking, receptor recycling, mitochondrial function and dopamine signaling [25]. In addition, it is currently not yet fully understood if VPS35 mutation leads to a loss or a gain of function of the retromer [26]. Another part of the retromer complex is the SNX-BAR heterodimers (Fig. 3a) composed of SNX1 or SNX2 in combination with SNX5 and SNX6 [27]. The composition of the SNX-BAR heterodimers plays a role in cargo-sorting [28]. Therefore, we hypothesized that SNX5 is involved in the trafficking of toxic αSyn species. This hypothesis was supported by the fact that knockdown of SNX6, the alternative to SNX5 in the SNX-BAR heterodimers, increased toxicity. This observation suggests that the effect of SNX5 knockdown is specific and not due to the knockdown of retromer components in general.

We then investigated the intracellular localization of exogenously added αSyn in our model and found that upon uptake, αSyn was co-localized with the TGN, suggesting transportation of αSyn to the TGN in our cell model. Other studies have also shown that αSyn co-localizes with the TGN in human astrocytes [29] and in neuroblastoma cells [30]. Furthermore, we observed fragmentation of the TGN as a consequence of αSyn overexpression or treatment with exogenous αSyn. This is consistent with other studies finding that treatment with prefibrillar αSyn aggregates leads to TGN fragmentation in an immortalized fibroblast cell line derived from monkey kidneys [31]. Furthermore, fragmentation of the TGN has been described in nigral neurons of PD patients [32]. Moreover, in other PD cell models, αSyn overexpression leads to Golgi fragmentation [33, 34].

Together, these findings emphasize that our cell model reflects what is observed in patients and supports the relevance of our findings in the present study. Interestingly, SNX5 knockdown prevented trafficking of αSyn to the TGN and its fragmentation, suggesting that SNX5 is a key regulator in the trafficking of toxic αSyn species. A previous study in a yeast model found that αSyn blocks the ER–Golgi transport, and that overexpression of the orthologue of Rab1, a protein needed for the docking of transport vesicles with the Golgi apparatus and thus improving the TGN function, protects dopaminergic neurons from αSyn-induced toxicity in yeast [35]. In line with this study, we could also show that impairment of the TGN is toxic for dopaminergic neurons.

While restoration of the ER–Golgi traffic is one possibility to prevent toxicity, our data suggest that redirection of harmful αSyn species away from the TGN by knocking down *SNX5* could also be a strategy to prevent αSyn-induced cell death. Interestingly, we previously showed that αSyn is degraded by macroautophagy and that not only stimulation of autophagy [8] but also bypassing macroautophagy by prevention of the formation of autophagosomes, could protect against αSyn-induced toxicity [7]. These emphasize that in some situations, the stimulation and the bypassing of a specific intracellular pathway can both be protective. Furthermore, in H4 neuroglial cells, knockdown of distinct Rab GTPases that are involved in αSyn trafficking prevents the formation of αSyn inclusions [36].

However, in other cells, including macrophages, SNX5 is essential for cell functions like micropinocytosis [37]. Furthermore, in mice, *SNX5* knockout leads to respiratory failure [38]. Therefore, complete depletion of SNX5 is likely to be detrimental for the whole mechanism. However, reduction of its activity or specific down-regulation in dopaminergic neurons still appears to be a promising approach to reducing the burden of αSyn-induced toxicity in PD.

## Conclusion

In summary, we performed a genome-wide siRNA screening and identified *SNX5* as the top hit. We found that SNX5 protein as part of the retromer complex is involved in αSyn trafficking. αSyn is taken up and transported to the TGN, leading to TGN fragmentation. Both could be ameliorated by knockdown of *SNX5*. On the other hand, SNX6 knockdown is toxic to the cells, suggesting that a distinct regulation of intracellular αSyn trafficking involves SNX-BAR heterodimers. Further investigation on αSyn trafficking could lead to the development of new therapeutic options to save neurons from αSyn-induced cell death. In particular, SNX5 seems to be a promising novel target for the development of a neuroprotective treatment for PD and related synucleinopathies.

## Supplementary Information

Additional file 1. **Figure S1**: Comparison of different siPOOL concentrations. **Figure S2**: Quantification of αSyn total intracellular and extracellular levels upon knockdown of SX5. **Figure S3**: Investigation of the effect of the knockdown of other SNXs. **Figure S4**: Brefeldin A (BFA) treatment led to Golgi fragmentation and cytotoxicity. **Figure S5**: The figure shows a version of panel c of panel 6 from the main manuscript with inclusion of the red channel that shows the signal of labeled αSyn. **Table S1**: Antibodies used for Western blot analysis. **Table S2**: Antibodies used for immunocytochemistry. **Supplementary methods** **of Brefeldin A treatment**.

Additional file 2. Sequences of used siPOOL siRNAs.

Additional file 3. **Figure S6**: Full Western blots shown in Fig. 2, Fig. 4, and Fig. S2.

## Data Availability

All datasets used and analyzed in this study are available from the corresponding authors on reasonable request.

## Abbreviations

αSyn: Alpha-synuclein
BAR: Bin/amphiphysin/Rvs
DAPI: 4′,6-Diamidino-2-phenylindole
esiRNAs: Endoribonuclease-prepared small interfering RNAs
GFP: Green fluorescent protein
GM: Growth medium
HBSS: Hanks' balanced salt solution
HRP: Horse radish peroxidase
LDH: Lactate dehydrogenase
NADH: Nicotinamide adenine dinucleotide
NHS: Normal horse serum
PD: Parkinson’s disease
PFA: Paraformaldehyde
PI: Propidium iodide
RNAi: RNA interference
ROI: Region of interest
SNX5: Sorting nexin 5
TGN: Trans Golgi network
VPS35: Vacuolar protein sorting ortholog 35
