# Supplementary material for: A genome-wide RNA interference screening reveals protectiveness of SNX5 knockdown in a Parkinson’s disease cell model
Source: Transl Neurodegener. 2025 Jun 3;14:27. doi: 10.1186/s40035-025-00486-5 (PMC12131658; doi:10.1186/s40035-025-00486-5)
Supplement: Supplementary file 1 — Additional file 1. Figure S1: Comparison of different siPOOL concentrations. Figure S2: Quantification of αSyn total intracellular and extracellular levels upon knockdown of SX5. Figure S3: Investigation of the effect of the knockdown of other SNXs. Figure S4: Brefeldin A (BFA) treatment led to Golgi fragmentation and cytotoxicity. Figure S5: The figure shows a version of panel c of panel 6 from the main manuscript with inclusion of the red channel that shows the signal of labeled αSyn. Table S1: Antibodies used for Western blot analysis. Table S2: Antibodies used for immunocytochemistry. Supplementary methods of Brefeldin A treatment. [file 40035_2025_486_MOESM1_ESM.pdf]

Supplementary Material

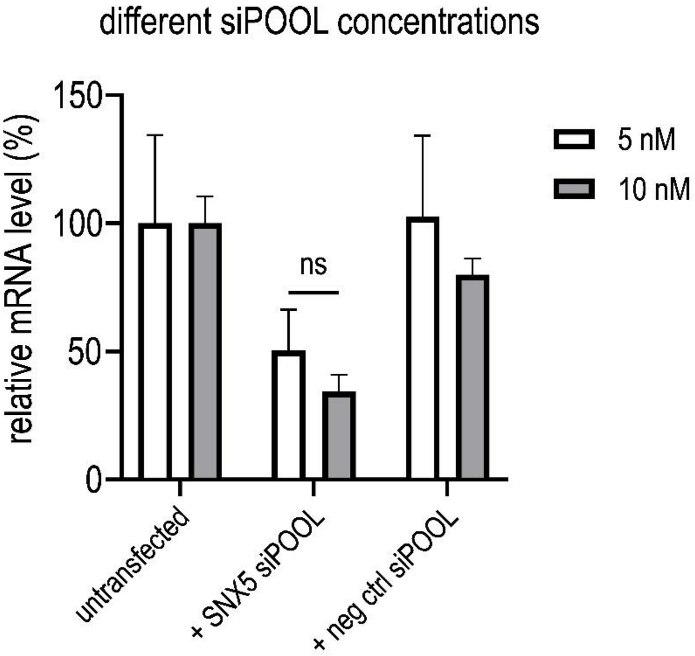

Supplementary Figure S1: Comparison of different siPOOL concentrations

Quantification of *SNX5* mRNA levels in control cells (without αSyn-overexpression) in untransfected cells, in cells transfected with 5nM siPOOL siRNA against SNX5, cells transfected with 10 nM siPOOL siRNA against SNX5 or cells transfected with negative control (neg ctrl) siPOOL siRNA. Transfection with 10 nM siPOOL siRNA did not significantly increased the knockdown (5nM: 49.5 ± 15.9% reduction in mRNA level; 10 nM: 65.8 ± 6.6% reduction in mRNA level).

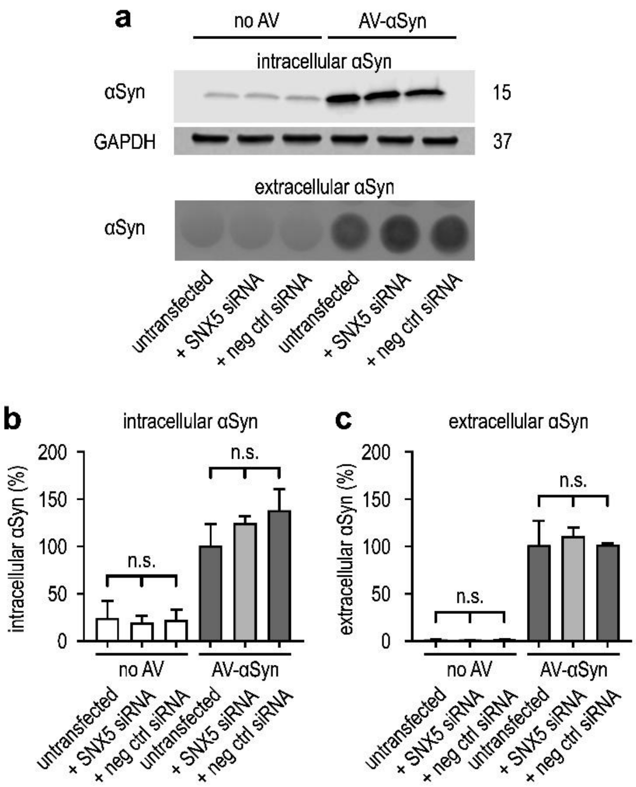

Supplementary Figure S2: Quantification of αSyn total intracellular and extracellular levels upon knockdown of SX5

a: Western blot with an antibody against αSyn from cell samples showing intracellar αSyn (top panel) and dot blot from the medium showing extracellular αSyn (bottom panel) from untransduced cells (no AV) and αSyn overexpressing cells (AV- αSyn). The cells were either untransfected or transfected with siRNA against SNX5 (SNX5 siRNA), or negative control siRNA (neg ctrl). The full Western blot is shown in Suppl. Fig. S6 f. **b**: Quantification of the Western blots representatively shown in **a** (top panel). Data are presented as mean ± SEM. n.s. not significant. **c**: Quantification of the dot blot analysis representatively shown in **a** (bottom panel). Data are presented as mean ± SEM. n.s. not significant.

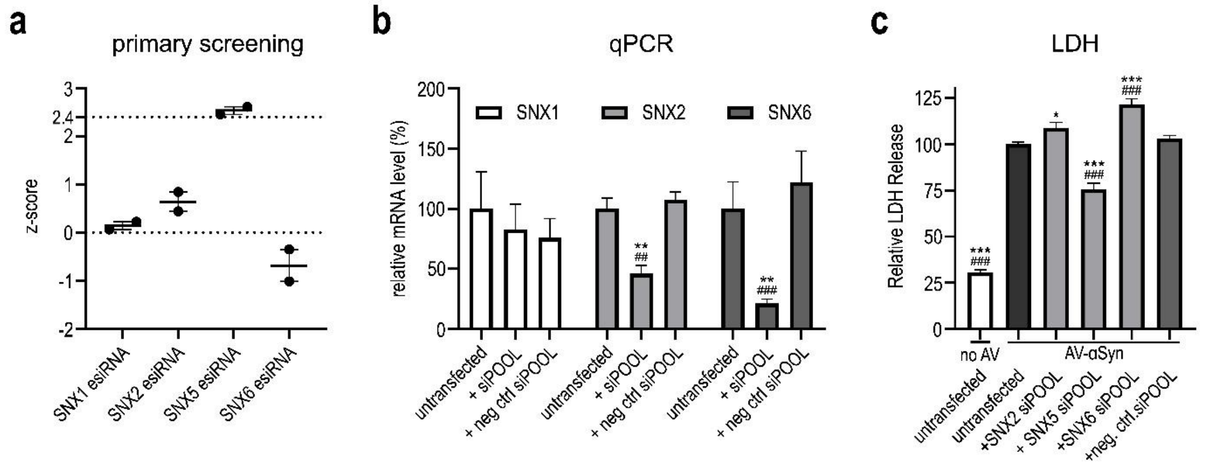

Supplementary Figure S3: Investigation of the effect of the knockdown of other SNXs

a: Results from the primary screening. In contrast to the knockdown of SNX5 (z -score: 2.54), the other proteins of the retromer complex (SNX1, SNX2 and SNX6) did not show a z-score > 2.4 and were therefore not selected as positive hits. Interestingly, the knockdown of SNX6 led to a z-score of -0.68, indicating a mild toxic effect of the knockdown. **b**: To validate the results from the primary screening, we quantified the mRNA levels of *SNX1*, *SNX2* and *SNX6* in control cells in untransfected cells, in cells transfected with the respective siPOOL siRNA or transfected with negative control (neg ctrl) siPOOL siRNA. The transfection with siPOOL siRNAs against SNX2 and SNX6 resulted in a significant reduction of the mRNA levels. **c:** LDH assay performed in untransduced control cells (no AV, white bar), untransfected αSyn-overexpressing cells (AV-αSyn; black bar), αSyn-overexpressing cells transfected with siPOOL siRNAs against SNX2, SNX5 or SNX6 (light grey bars) and αSyn-overexpressing cells transfected with negative control (neg ctrl) siPOOL siRNA (dark grey bar). The knockdown of SNX6 led to a significant increase of LDH release, confirming the result from the primary screen. In the LDH assay, the knockdown of SNX2 also led to a significant higher LDH release. In contrast, the knockdown of SNX5, as shown before, has a protective effect on the cells, indicated by the reduced LDH release.

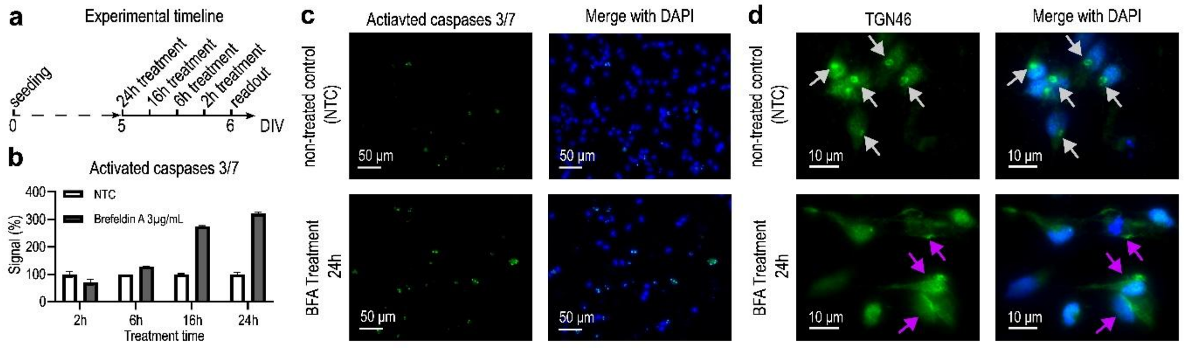

Figure S4: Brefeldin A (BFA) treatment led to Golgi fragmentation and cytotoxicity

a: Experimental timeline of the Brefeldin A (BFA) treatment experiments. **b:** Quantification of the CellEvent™ signal, a staining for activated caspases 3 and 7, in cells treated with 3µg/mL BFA at different timepoints (2h, 6h, 16h or 24h before analysis). 16h and 24h treatment led to a higher activation of caspases 3 and 7 indicating a toxic effect of the BFA treatment. **c:** Representative images of the CellEvent™ staining (left side images) without treatment (NTC) or after 24h treatment. The DAPI-merged images are shown on the right side. **d:** Cells without treatment (NTC) or cells treated for 24h with BFA were stained for the golgi marker TGN46 (left side images). The DAPI-merged images are shown on the right side. The white arrows indicate the ring-like structure of the non-fragmented TGN in non-treated cells. In contrast, the TGN is fragmented in the BFA treated cells (purple arrow).

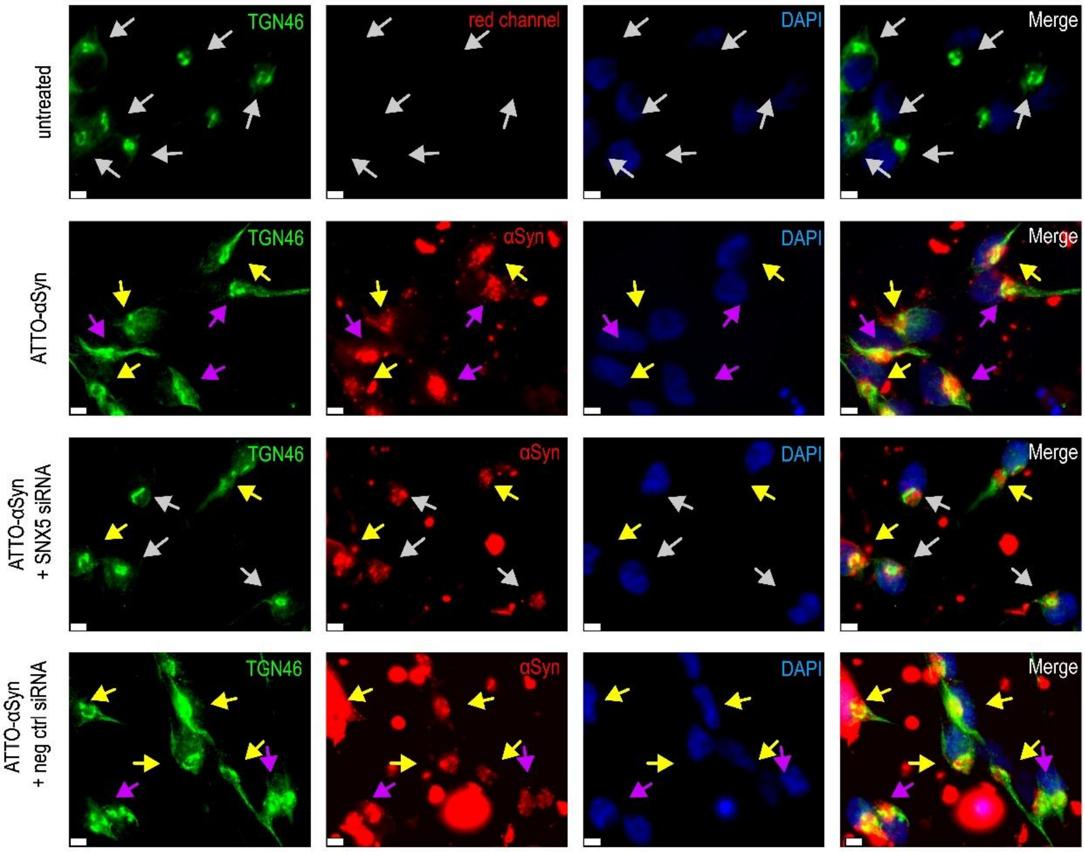

Supplementary Figure S5: The figure shows a version of panel c of panel 6 from the main manuscript with inclusion of the red channel that shows the signal of labeled αSyn.

Supplementary Table S1: Antibodies used for Western blot analysis:

Antibodies for WB	Product Number	Company	Dilution
SNX5	sc515215	Santa Cruz	1:1000
GAPDH	2118S	Cell Signalling	1:2000
VPS35	ab10099	abcam	1:1000
SNX2	sc390510	Santa Cruz	1:1000
SNX6	sc36565	Santa Cruz	1:1000
SNX1	sc376376	Santa Cruz	1:1000
αSyn	701085	Invitrogen	1:750
LAMP2a	ab18528	abcam	1:200
LAMP2	MA1-205	Invitrogen	1:1000
p62	MABC32	Merck Millipore	1:1000
LC3B	3868S	Cell Signalling	1:1000
β-actin	1028165	Sigma-Aldrich	1:2000
Secondary antidodies			
HRP Horse anti-mouse IgG	PI-2000	Thermo Fisher Scientific	1:2500
HRP Goat anti-rabbit IgG	PI-1000	Thermo Fisher Scientific	1:2500

Supplementary Table S2: Antibodies used for immunocytochemistry

Antibodies for ICC	Product Number	Company	Dilution
αSyn	sc-12767	Santa Cruz	1:1000
TGN46	ab50595	abcam	1:200-1:1000
Rab 5a	sc-16660	Santa Cruz	1:100
Rab7	ab137029	abcam	1:100
LAMP1	ab25630	abcam	1:1000
p62	ab91526	abcam	1:50
LC3B	3868S	Cell Signalling	1:400
LAMP2a	ab18528	abcam	1:200
Rab11a	3H18L5	Invitrogen	1:1000
Secondary antidodies			
488 Donkey anti-mouse	A21202	Thermo Fisher Scientific	1:1000
488 Donkey anti-rabbit	A21206	Thermo Fisher Scientific	1:1000
594 Donkey anti-mouse	A21203	Thermo Fisher Scientific	1:1000
594 Donkey anti-rabbit	A21207	Thermo Fisher Scientific	1:1000

Supplementary methods:

Brefeldin A treatment

LUHMES cells were cultured as described in the material and methods section. The known Golgi disturbing agent Brefeldin A (BFA, Thermo Fisher Scientific; Dinter et al. 1998) was used to show the cytotoxic effect of the TGN fragmentation. Therefore, the cells were treated at different timepoints with 3 µg/mL BFA as it is shown in the experimental timeline. ON DIV6, the cells were either stained for activated caspase 3/7 with the CellEvent™ staining or immunostained with an antibody against TGN46 to visualize the TGN fragmentation.

References:

Dinter A, Berger EG. Golgi-disturbing agents. Histochem Cell Biol. 1998 May-Jun;109(5-6):571-90. doi: 10.1007/s004180050256. PMID: 9681636.
