# Supplementary material for: A genome-wide RNA interference screening reveals protectiveness of SNX5 knockdown in a Parkinson’s disease cell model
Source: Transl Neurodegener. 2025 Jun 3;14:27. doi: 10.1186/s40035-025-00486-5 (PMC12131658; doi:10.1186/s40035-025-00486-5)
Supplement: Supplementary file 3 — Additional file 3. Figure S6: Full Western blots shown in Fig. 2, Fig. 4, and Fig. S2. [file 40035_2025_486_MOESM3_ESM.pdf]

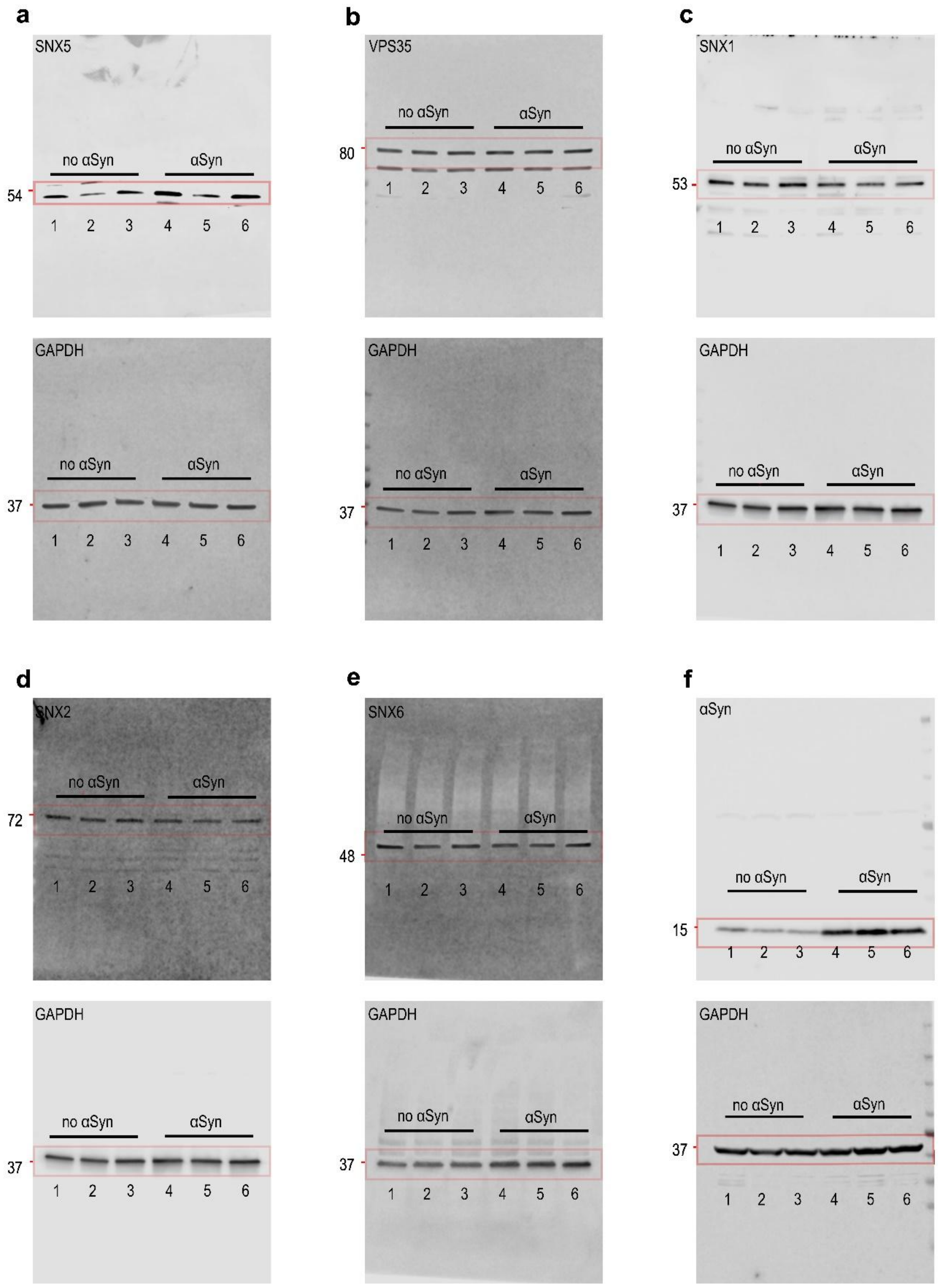

Supplementary Figure S6 full Western blots shown in Fig. 2, Fig. 4, and Suppl. Fig. S2

a: The panel shows the full Western blots from Fig. 2 c. The upper panel shows the full Western blot after staining with an SNX5 antibody, the lower panel shows the full Western blot after staining with an antibody against GAPDH used as loading control, b-e: The panal show the full Western blots from Fig 4 b. The upper panels show the Western blots after staining with antibodies against VPS35 (b), SNX1 (c), SNX2 (d), SNX6 (e), the lower panels show the respective with an antibody against GAPDH used as loading control. f: The panels show the full Western blot from Suppl. Fig. S2. The upper panel

shows the Western blot after a staining with an antibody against alpha-synuclein. The lower panel shows the Western blot after a staining with an antibody against GAPDH used as loading control. In all panels, the red frame marks the cutout that is shown in the related figures. The conditions are the same in all Western blots: 1: untransduced cells without siRNA transfection, 2: untransduced cells after transfection with SNX5 siPOOL siRNA, 3: untransduced cells after transfection with a negative control siPOOL siRNA, 4: αSyn-overexpressing cells without siRNA transfection, 5: αSyn-overexpressing cells after transfection with SNX5 siPOOL siRNA, 6: αSyn-overexpressing cells after transfection a negative control siPOOL siRNA.
